# Sustainable Photocatalysis with Phenyl-Modified g-C_3_N_4_/TiO_2_ Polymer Hybrids: A Combined Computational and Experimental Investigation

**DOI:** 10.3390/polym17101331

**Published:** 2025-05-14

**Authors:** Riccardo Dettori, Sahar Aghapour Ghourichay, Stefania Porcu, Claudio Melis, Luciano Colombo, Pier Carlo Ricci

**Affiliations:** Department of Physics, University of Cagliari, 09042 Monserrato, Ca, Italystefania.porcu@dsf.unica.it (S.P.); claudio.melis@dsf.unica.it (C.M.); luciano.colombo@dsf.unica.it (L.C.); carlo.ricci@unica.it (P.C.R.)

**Keywords:** polymer photocatalyst, photocatalysis, carbon nitride, TiO_2_, rutile, heterostructure, DFTB

## Abstract

We combined atomistic simulations and experiments to assess the photocatalytic potential of the rutile phase of ${\mathrm{TiO}}_{2}$ combined with phenyl-modified carbon nitride (PhCN). Density Functional Tight Binding (DFTB) calculations predict favorable adhesion properties and type-II band alignment, crucial for efficient charge separation between PhCN and rutile TiO_2_ surfaces. These theoretical predictions are validated experimentally: structural (XRD and Raman) and optical characterizations confirm the successful formation of a PhCN/rutile hybrid and indicate beneficial electronic interactions. Importantly, photocatalytic tests under visible light reveal significant degradation activity, confirming that the computationally predicted synergistic effects render the PhCN/rutile system a promising, potentially greener alternative to traditional anatase-based photocatalysts.

## 1. Introduction

Due to its exceptional structural and optoelectronic properties, graphitic carbon nitride (g-$C_{3}N_{4}$) has emerged as a promising material for photocatalytic applications [1,2]. Its two-dimensional layered framework comprises tri-s-triazine (or heptazine) units linked by nitrogen bridges, forming a robust polymeric network stabilized by van der Waals forces. This distinctive configuration imparts several key advantages, including excellent thermal and chemical stability, biocompatibility, and eco-friendliness. The π-conjugated planar structure enhances charge carrier mobility. At the same time, its moderate bandgap (∼2.7 eV) enables efficient absorption of visible light, making it a good candidate for solar energy conversion and other light-driven applications [3]. Building on these peculiar properties, g-$C_{3}N_{4}$ has shown great potential over the years in diverse fields such as photocatalytic water splitting, ${\mathrm{CO}}_{2}$ reduction, environmental remediation, organic synthesis, and energy harvesting [3]. Despite the potential of this material, its practical applications are limited by its high electron–hole recombination rates and restricted absorption within the visible spectrum. To overcome these challenges, significant research efforts have been made using g-$C_{3}N_{4}$ with other semiconductors to develop hybrid systems [4,5,6]. Among these, g-$C_{3}N_{4}$ and titanium dioxide (${\mathrm{TiO}}_{2}$) are a particularly effective combination, offering improved photocatalytic performance and expanded light absorption capabilities [1,7]. ${\mathrm{TiO}}_{2}$ is a well-established photocatalyst known for its excellent photocatalytic activity, high chemical stability, and non-toxic nature. However, its wide bandgap (3.2 eV for the anatase phase) limits its light absorption primarily to the ultraviolet (UV) region, which accounts for only about 5% of the solar spectrum. When ${\mathrm{TiO}}_{2}$ is combined with graphitic carbon nitride, the resulting heterostructure effectively integrates the complementary properties of the two materials. ${\mathrm{TiO}}_{2}$ provides a stable platform for charge separation and transport. At the same time, g-$C_{3}N_{4}$ broadens the light absorption range into the visible spectrum. This synergistic interaction in the heterojunction promotes efficient separation of photogenerated electron–hole pairs, significantly enhancing photocatalytic performance [8]. Furthermore, the development of phenyl-modified carbon nitride (PhCN) [9,10,11] represented a significant breakthrough: incorporating phenyl groups extends the π-conjugation within the framework, effectively reducing its bandgap and shifting its light absorption deeper into the visible spectrum. It should be noted here that the commercial P25 (−80% anatase, −20% rutile) photocatalyst possesses a very efficient charge separation mechanism, which results in enhanced formation of OH radicals on the nanoparticle surface. Several recent studies were dedicated to the synthesis and formation of heterostructures based on P25 and g-$C_{3}N_{4}$, combining the excellent photocatalytic properties of commercial ${\mathrm{TiO}}_{2}$ with the charge transfer mechanism from g-$C_{3}N_{4}$. However, the reactions producing P25/g-$C_{3}N_{4}$ hybrids generally require post-mixing or high-temperature calcination steps that can degrade the organic scaffold [12,13,14]. In addition, PhCN significantly improves light-harvesting efficiency and enhances charge transport properties [9]. Upon light excitation, electrons transitioning from the highest occupied to lowest unoccupied molecular orbitals (HOMO and LUMO) of PhCN are transferred to the ${\mathrm{TiO}}_{2}$ conduction band, leading to higher rates of pollutant degradation under visible light. Under visible-light irradiation, the degradation efficiency of Rhodamine B solutions increased from 17% with g-$C_{3}N_{4}$/${\mathrm{TiO}}_{2}$ to 98% with the PhCN/${\mathrm{TiO}}_{2}$ hybrid system. This substantial improvement highlights the critical role of phenyl modification in optimizing photocatalytic activity [15].

In this work, we further improve the hybrid system by replacing the anatase phase of ${\mathrm{TiO}}_{2}$ with the rutile phase as a more sustainable and cost-effective alternative. Rutile offers significant advantages in terms of simpler and greener synthesis methods. For instance, replacing ethanol with water as a solvent during the synthesis process aligns with the principles of green chemistry [16,17,18]. This eco-friendly approach minimizes the environmental impact and reduces production costs, making the PhCN/${\mathrm{TiO}}_{2}$ hybrid system a more practical and sustainable solution. Here, we explore the effectiveness of this strategy through a combined computational and experimental approach. Density Functional Tight Binding (DFTB) calculations are employed to investigate the electronic properties, band alignment, and adsorption energetics of PhCN/${\mathrm{TiO}}_{2}$ heterostructures. The results reveal that (i) the adhesion properties between PhCN and the rutile phase are comparable to those observed between anatase and pristine g-$C_{3}N_{4}$, and (ii) the band alignment indicates strong potential for photocatalytic applications. All computational findings are paralleled with experimental Raman, X-ray diffraction (XRD), diffuse reflectance spectroscopy, and photocatalytic degradation experiments. Specifically, Raman and XRD characterizations confirm the effective formation of the anatase and rutile structures, as well as the successful integration of g-$C_{3}N_{4}$ and PhCN, with adhesion observed in both phases. Optical characterization further demonstrates interactions between the organic and inorganic components, highlighting the charge transfer process from the organic moiety, which acts as a sensitizer, to ${\mathrm{TiO}}_{2}$. Finally, photocatalytic degradation experiments validate the predicted charge transfer behavior and photocatalytic efficiency, identifying PhCN/rutile as a promising green photocatalyst.

## 2. Materials and Methods

### 2.1. Computational Methods

This study adopts DFTB calculations to investigate the electronic properties and band alignment of PhCN/${\mathrm{TiO}}_{2}$ heterostructures. DFTB is a semi-empirical quantum mechanical method derived from Density Functional Theory (DFT) that simplifies the Kohn–Sham equations by employing a minimal localized basis set and precomputed integrals [19,20,21]. This approach retains the essential physics of DFT while significantly reducing computational cost, enabling the simulation of large systems with hundreds of atoms that would be computationally prohibitive using standard DFT methods. Such efficiency is crucial for modeling complex interfaces between extended ${\mathrm{TiO}}_{2}$ surfaces and sizable organic molecules such as triazine and heptazine. While DFT offers high accuracy for electronic structure calculations, its computational demands scale poorly with system size. DFTB provides a balance between accuracy and efficiency and has been successfully applied to modeling both gas-phase molecules [22] and condensed matter under inert and reactive conditions [23,24,25,26], including extreme pressures and temperatures [27,28].

The DFTB total energy is derived from an expansion of the Kohn–Sham energy to the second or third order in charge fluctuations, yielding the expression

(1) $$E_{\mathrm{DFTB}}=E_{\mathrm{BS}}+E_{\mathrm{Coul}}+E_{\mathrm{rep}}$$

where $E_{\mathrm{BS}}$ is the band structure energy, $E_{\mathrm{Coul}}$ is the charge fluctuation term, and $E_{\mathrm{rep}}$ is the repulsive energy. The band structure energy is calculated as a sum over occupied electronic states from the DFTB Hamiltonian. The Hamiltonian matrix elements are determined from pre-tabulated Slater–Koster tables derived from reference calculations with a minimal basis set. The on-site matrix elements are the free-atom orbital energies, and the off-site terms are computed using a two-center approximation, where both wavefunctions and electron density are subjected to confining potentials. The repulsive energy, instead, accounts for ion–ion repulsions as well as Hartree and exchange-correlation double-counting terms. This term is typically expressed as an empirical function with parameters fitted to reproduce high-level quantum mechanical or experimental reference data. In addition, a dispersion correction can be included, such as those commonly used in DFT calculations [29,30]. To model $E_{\mathrm{rep}}$ in our simulations, we employed a combination of publicly available DFTB parameter sets: *mio-1-1* [19,31] and *tiorg-0-1* [32]. These parameter sets have been developed for organic molecules containing O, N, C, H, and S, as well as for bulk titanium oxide, titanium oxide surfaces, and the interactions of titanium oxide with organic molecules within the DFTB framework.

Reference DFT simulations were performed using the Vienna Ab initio Simulation Package (VASP) [33,34,35], employing the projector augmented wave (PAW) method [36,37] and the Perdew–Burke–Ernzerhof (PBE) exchange-correlation functional [38]. Partial occupancies of the electronic states were set using fourth-order Methfessel–Paxton smearing [39] with a width of 0.05 eV. Converged energies for the bulk systems were achieved with a plane-wave energy cutoff of 600 eV and a self-consistent field (SCF) convergence criterion of $10^{-8}$ eV. The force convergence tolerance was set to 0.01 eV/Å for each atom in all directions. DFTB calculations were performed using the DFTB+ code [20], employing orbital-resolved self-consistent charge (SCC) calculations [19]. The same convergence criteria and electron thermal smearing as in the DFT calculations were adopted. In DFTB, the total energy expression with SCC is derived by assuming spherically symmetric charge densities and expanding the Kohn–Sham total DFT energy expression to the second [19] or third order [21] in charge fluctuations; in this work, we rely exclusively on the second-order charge fluctuation model. A k-point Monkhorst–Pack mesh sampling of the Brillouin zone [40] was used: for bulk anatase, a 10×10×4 mesh was considered, while for bulk rutile, we adopted a 8×8×12 mesh. Regarding the slabs, a 2×2×1 mesh was used for anatase-derived slabs and a 3×2×1 mesh for rutile slabs.

#### Slab Generation and Assessment of Simulation Parameters

A series of model systems was constructed for both the anatase and rutile phases of ${\mathrm{TiO}}_{2}$. Two crystallographic orientations were considered for each phase: the (100) and (110) surfaces. These surfaces were selected due to their distinct atomic arrangements and surface energies, which can significantly influence adsorption behavior and electronic interactions with organic molecules [41,42]. The bulk structures of anatase and rutile ${\mathrm{TiO}}_{2}$ were first optimized using DFT calculations to obtain accurate lattice parameters. A comparison between DFT- and DFTB-calculated lattice constants is reported in Table 1.

Overall, the DFTB lattice constants show good agreement with the DFT results and experimental data [43], confirming the reliability of our computational approach. Furthermore, the calculated band gaps (3.0 eV for anatase and 2.8 eV for rutile) are consistent with experimental values of 3.2 eV and 3.0 eV, respectively [44]. Using these optimized bulk structures, slab models were generated for the (100) and (110) surfaces of both the anatase and rutile phases. The slabs were constructed by cleaving the bulk crystals along the respective crystallographic planes, resulting in surfaces with specific atomic terminations. We chose the ones with the lowest energy among all possible terminations. Each slab consisted of three atomic layers, which provides a balance between capturing the representative properties of the bulk material and achieving computational efficiency. A vacuum layer of 15 Å was included perpendicular to the surface to prevent interactions between periodic images in the slab model. To accommodate the adsorption of the organic molecules considered in this study without introducing significant strain or artificial interactions, supercells were constructed by replicating the slabs in the in-plane directions. Specifically, a 2×2 supercell was used for anatase slabs, and a 3×2 supercell was used for rutile slabs. As a further check for the validity of our parameter sets, we computed the formation energy for the obtained surfaces. We compared them with values available in the literature, as presented in Table 2. A complete collection of the atomistic views for the structures investigated herein (both bulk and slab systems) is shown in Figure S1 of the Supporting Information (SI).

Our findings compare very well with the range of values reported in the literature for anatase surfaces, while the formation energies are somewhat overestimated for rutile. However, we remark that the formation energies are highly dependent upon the choice of simulation parameters and functional, thus the observed offset is not a significant issue here. These observations further support the validity of the adopted computational methodology.

### 2.2. Experimental Methods

Phenyl-triazine, melamine, Rhodamine B (RhB 95%), and absolute ethanol were purchased from Merck/Sigma-Aldrich (Darmstadt, Germany) and Carlo Erba (Val de Reuil, France), respectively.

Preparation of g-$C_{3}N_{4}$

Graphitic carbon nitride (g-$C_{3}N_{4}$) was synthesized through the thermal polymerization of melamine. Specifically, 1 g of melamine was placed in a crucible and heated in a furnace at 550 °C for 4 h under static air conditions.

Preparation of phenyl-modified carbon nitride

Phenyl-modified carbon nitride was synthesized by placing 1 g of 6-phenyl-1,3,5-triazine-2,4-diamine powder in a quartz tube, which was then positioned in a tubular furnace. The material was subjected to a controlled heating process, with the temperature gradually increased to 400 °C over 1 h.

Preparation of PhCN/TiO_2_ in ethanol

A total of 100 mg of hCN was dispersed in 20 mL of ethanol and stirred at room temperature for 30 min to ensure uniform dispersion. Subsequently, 0.5 mL of titanium tetrachloride (TiCl_4_) was added dropwise to the solution, stirring continuously for an additional 2 h. The resulting mixture was transferred to an autoclave and subjected to hydrothermal treatment at 180 °C for 8 h. Following the reaction, the product was filtered, washed with ethanol to remove impurities, and dried. Finally, the dried sample was ground into a fine powder for subsequent characterization.

Preparation of PhCN/TiO_2_ in water

In this case, we used wet-chemical and sol-gel synthesis methods. Initially, 100 mg of PhCN was dispersed in 20 mL of deionized water and stirred at room temperature for 30 min to form a homogeneous suspension. Then, 0.5 mL of titanium tetrachloride (TiCl_4_) was added dropwise to the mixture, followed by continuous stirring for another 2 h to promote interaction between the components. The resulting product was separated by filtration, thoroughly washed with deionized water to remove unreacted residues, and dried at 60 °C. Finally, the dried sample was ground into a fine powder to obtain the final material.

Preparation of g-$C_{3}N_{4}$/${\mathrm{TiO}}_{2}$ in water

The same methods (wet chemical and sol–gel) used for synthesizing PhCN/${\mathrm{TiO}}_{2}$ in water were applied.

Characterization

XRD analysis was conducted at room temperature using a Rigaku Miniflex II diffractometer equipped with Cu K_*α*_ radiation (λ=1.54118Å) in a θ−2θ Bragg–Brentano geometry. Diffuse reflectance spectroscopy was used to measure the samples’ absorption properties, employing a UV–Vis–NIR JASCO FP-8550ST spectrometer (Jasco, Easton, MD, USA) equipped with a PbS solid-state photodetector. The measurements were carried out in a reflection configuration, with the diffuse reflectance compared against a BaSO_4_ reference. Absorption features were determined using the Kubelka–Munk equation. The Raman spectra were recorded using a Sol Instruments MS750 series monochromator–spectrograph (Sol Instruments, Augsburg, Germany). An excitation wavelength of 785 nm was employed for ${\mathrm{TiO}}_{2}$ samples, while 1064 nm was used for the hybrid systems, with a spectral resolution of approximately 1 $\;{\mathrm{cm}}^{-1}$.

Photodegradation of Rhodamine B

The photocatalytic performance was tested by measuring the degradation of Rhodamine B (RhB) in an aqueous solution under visible light. A Philips 13 W white LED light source (100 mW optical power) was used for irradiation. To ensure equilibrium between the catalyst and the dye, 40 mg of the catalyst was mixed with 40 mL of a 10 mg/L RhB solution and stirred in the dark for 30 min. Afterward, the mixture was exposed to visible light. During the reaction, 1.5 mL samples were taken every 60 min. These samples were centrifuged to separate the catalyst, and the remaining RhB concentration was measured using a Jasco V-750 spectrophotometer with a spectral bandwidth of 2nm in the 200–800nm range, monitoring the maximum at 554 nm.

## 3. Results and Discussion

### 3.1. Computational Results: Stability and Energetics of the Heterostructure

As mentioned in Section 2, the preparation of g-$C_{3}N_{4}$ typically involves the thermal condensation of nitrogen-rich organic precursors; triazine and heptazine are recognized as such precursors [47,48] and serve as fundamental building blocks of graphitic carbon nitride. Given the large spatial dimensions of a typical g-$C_{3}N_{4}$ sample, the computational workload would be prohibitive even for DFTB calculations. Therefore, we opted to study the band alignment properties of triazine and heptazine with ${\mathrm{TiO}}_{2}$. The extended electronic properties of polymeric systems such as g-$C_{3}N_{4}$ are often dominated by the characteristics of their essential components. In the case of g-$C_{3}N_{4}$, triazine and heptazine units largely determine the frontier orbital distribution and, hence, the band edges that govern photocatalytic and charge-transfer processes. Previous theoretical studies have shown that the highest occupied and lowest unoccupied molecular orbitals (HOMO and LUMO) in g-$C_{3}N_{4}$ are primarily localized on these units [49,50]. In addition, recent studies have shown that doping carbon nitride-based materials with phenyl rings can decrease the bandgap and increase the separation rate of electron–hole pairs [51]. For this reason, we decided to compare triazine and heptazine with their phenyl-functionalized counterparts (Ph-triazine and Ph-heptazine). The molecules investigated in this work are shown in Figure S2 (SI).

**Figure 1:**
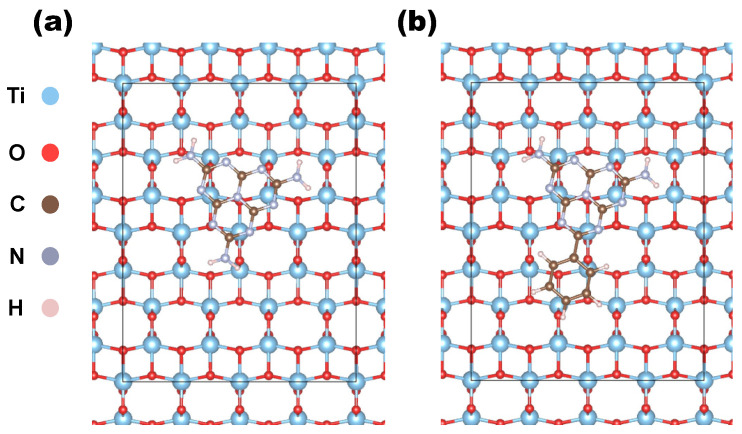
(**a**) Top of a heptazine molecule on the (100) anatase slab; (**b**) top view of Ph-heptazine on the same facet.

The organic molecules were initially fully relaxed in vacuum conditions and then placed atop the constructed ${\mathrm{TiO}}_{2}$ slabs, with their principal planes parallel to the surface and randomly oriented in the in-plane directions. The resulting organic molecule–substrate complexes were fully optimized according to the aforementioned convergence criteria. Figure 1 illustrates two different views of heptazine and phenyl-heptazine deposited on the (100) surface of anatase ${\mathrm{TiO}}_{2}$. We generated a total of 16 heterostructures by varying the deposited molecule, the surface orientation, and the phase of the substrate. The computed adsorption energies for these heterostructures are reported in Table 3.

The adsorption energies indicate that adsorption on the (110) facet is stronger than on the (100) facet, suggesting that chemisorption may occur on the former. At the same time, the interaction on the latter is predominantly a physisorption phenomenon, dominated by van der Waals forces and weak electrostatic interactions. The (110) surface orientation has a higher density of unsaturated surface atoms and undercoordinated sites [52], which enhance interactions with adsorbed molecules through stronger chemical bonding or increased van der Waals forces. In comparing the role of the substrate, we observe that, across all molecules and surface orientations, the adsorption energies are more negative on anatase surfaces than on rutile surfaces. Anatase typically exhibits higher photocatalytic activity and more reactive surface sites due to its electronic structure and surface energy [53]. Specifically, the anatase phase presents more undercoordinated Ti atoms and oxygen vacancies, which can form stronger bonds with adsorbates. Heptazine molecules exhibit slightly more negative adsorption energies for most surfaces than triazine, especially on the anatase (110) surface. This trend may be related to the more extensive conjugated ring system of heptazine compared to triazine, which provides a greater area for interaction with the surface. The extended interaction area enhances π−d orbital interactions between the delocalized electrons of the organic molecule and the *d*-orbitals of Ti atoms. Finally, phenyl-functionalized molecules exhibit more negative adsorption energies on the (110) surfaces than their pristine counterparts. The addition of a phenyl group increases the molecular size and introduces additional π electrons, which can enhance π−π stacking interactions with the ${\mathrm{TiO}}_{2}$ surface and increase van der Waals forces, especially on surfaces with higher atomic density, such as the (110) facet.

These results show that phenyl functionalization enhances triazine and heptazine adhesion properties. Furthermore, moving from anatase to rutile, both heptazine and triazine—though with slightly reduced strength—still adhere to the ${\mathrm{TiO}}_{2}$ surface. In all cases, the preferred adsorption configuration remains the face-on orientation.

### 3.2. Computational Results: Band Alignment

**Figure 2:**
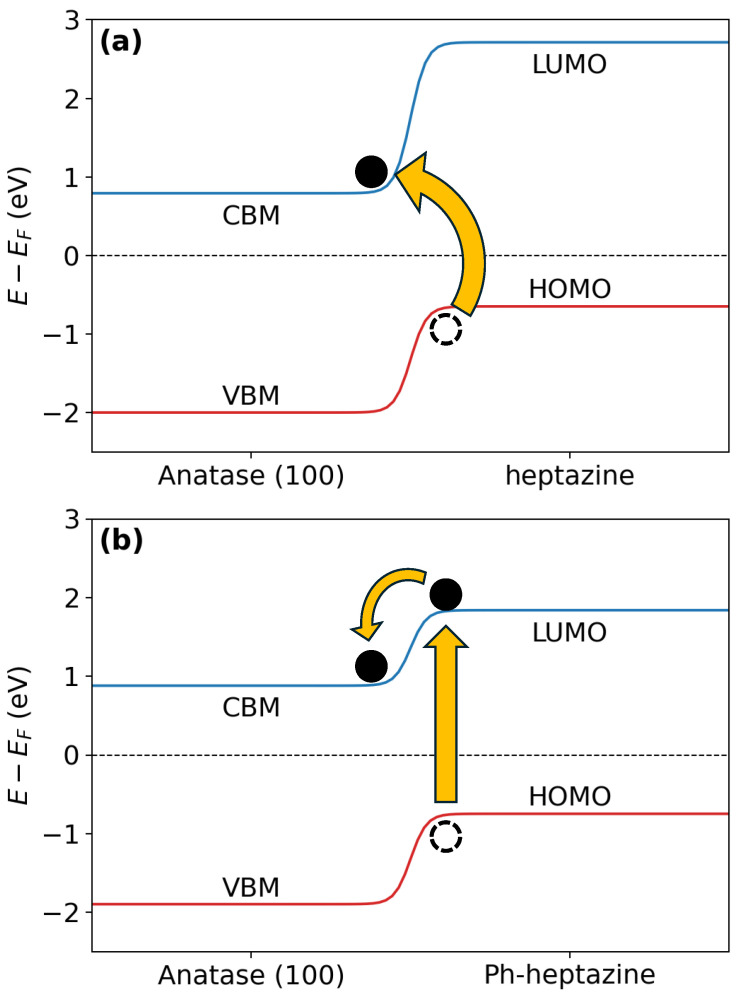
Schematic representation of the band alignment along with the proposed electron transfer mechanism for (**a**) triazine on (100)-anatase and (**b**) Ph-triazine on the same substrate.

To calculate the band alignment, we performed a detailed analysis of the electronic density of states (DOS) for the fully relaxed heterostructures. The total electronic density of states (DOS) was projected onto the atomic species to separate the contributions from the ${\mathrm{TiO}}_{2}$ substrate and the adsorbed organic molecules to identify the specific electronic states near the Fermi level ($E_{F}$). In determining the top of the valence band (VBM) and the bottom of the conduction band (CBM) for ${\mathrm{TiO}}_{2}$, as well as the HOMO and LUMO of the adsorbed molecules, a threshold criterion based on the normalized DOS was applied. Specifically, energy levels were defined as the energies at which the normalized DOS $>10^{-5}$ above and below $E_{F}$. This threshold ensured that only the relevant electronic states near the bandgap would be considered, isolating the significant electronic states contributing to interface charge transfer processes. As illustrated in Figure 2, this approach allows a clear visualization of how the energy levels of the ${\mathrm{TiO}}_{2}$ substrate align with those of the adsorbed molecules, providing insights into the potential for efficient charge separation and transfer. The complete set of projected DOS and band alignment results is provided in the SI.

The results of our calculations are rationalized by quantifying the energy difference between the CBM and the LUMO, $\Delta =E_{\mathrm{LUMO}}-E_{\mathrm{CBM}}$, and the difference between the CBM and the HOMO, $\Delta^{\prime }=E_{\mathrm{CBM}}-E_{\mathrm{HOMO}}$. These values are reported in Table 4.

Consistent with the observations in Figure 2, all heterostructures exhibit a type II (staggered gap) band alignment, as proven by Δ>0: the LUMO is higher in energy than the ${\mathrm{TiO}}_{2}$ CBM. This alignment promotes efficient charge separation and transfers across the interface, which are essential for enhancing device performance [54,55]. In particular, the phenyl-functionalized versions of the organic molecules consistently exhibit smaller Δ values than their pristine counterparts. As already pointed out when rationalizing the adsorption energies, adding a phenyl group extends the π-conjugation of the molecule, lowering its LUMO energy level. This shift brings the LUMO closer to the CBM, enhancing electron transfer. The stronger adsorption of phenyl-functionalized molecules suggests improved electronic interaction and orbital overlap, increasing charge transfer efficiency. Similarly, smaller Δ values are observed for heptazine than for triazine. The more extensive conjugated system of heptazine lowers its LUMO energy level relative to triazine, enhancing electronic interactions with ${\mathrm{TiO}}_{2}$ [56]. Anatase tends to show lower Δ values than rutile; the electronic structure of anatase ${\mathrm{TiO}}_{2}$ has its CBM at a higher energy level than rutile, reducing the energy gap with the molecule LUMO and promoting electron transfer. This trend correlates with the interaction energies, as anatase is characterized by stronger adsorption, likely due to higher proportions of undercoordinated Ti atoms and reactive sites than rutile. Finally, considering the surface orientation, it is observed that the (110) orientation is characterized by lower Δ values, particularly for phenyl-functionalized molecules adsorbed on anatase. Combining surface reactivity and molecular design leads to enhanced photocatalytic properties [57]. A reduced energy gap between the CBM and the LUMO facilitates efficient electron transfer from the molecule to the substrate upon photoexcitation [58,59].

$\Delta^{\prime }$ helps in quantifying the potential for direct electron transfer from the HOMO of the molecule to the CBM of ${\mathrm{TiO}}_{2}$ upon photoexcitation. This quantity provides a crucial insight into an additional charge transfer pathway, complementing the Δ values, which primarily focus on transitions involving the LUMO. Table 4 indicates that, in almost all cases, functionalization with the phenyl group leads to an increase in $\Delta^{\prime }$. In contrast, pristine triazine and heptazine molecules generally exhibit smaller $\Delta^{\prime }$ values, suggesting that, for phenyl-functionalized molecules, an indirect charge transfer from the LUMO of the molecule to the CBM of ${\mathrm{TiO}}_{2}$ is favored. Hence, in pristine triazine or heptazine molecules, a direct transfer from the HOMO to the CBM of ${\mathrm{TiO}}_{2}$ appears to be more likely. These two electron transfer mechanisms are shown schematically in Figure 2. The analysis of the band alignment suggests that, in both anatase and rutile, the addition of organic molecules extends absorption into the visible region. In fact, the reduction in the LUMO–HOMO difference effectively decreases the bandgap compared to that of ${\mathrm{TiO}}_{2}$. Furthermore, incorporating a phenyl group further reduces the HOMO–LUMO gap, resulting in a further redshift of the absorption spectrum toward the visible range. Our analysis also confirms that the staggered band alignment is achieved for both anatase and rutile, enabling efficient charge transfer from the organic molecule to ${\mathrm{TiO}}_{2}$.

Incorporating the phenyl group significantly reduces the value of $\Delta^{\prime }$, suggesting differing charge transfer mechanisms. In systems without the phenyl group, direct electron transfer from the HOMO of the molecule to the conduction band of ${\mathrm{TiO}}_{2}$ is expected. In contrast, in phenyl-functionalized systems, an indirect transfer mechanism is likely, where the electron first transitions to the LUMO of the molecule after photoexcitation and subsequently transfers to the conduction band of ${\mathrm{TiO}}_{2}$.

### 3.3. Experimental Results: Raman and XRD Measurements

These theoretical predictions were validated through a series of measurements (i) assessing the effective formation of the hybrid systems with the organic polymers on ${\mathrm{TiO}}_{2}$ surfaces using Raman spectroscopy and XRD analysis and (ii) analyzing the kinetic behavior of the excited systems using time-resolved measurements of the hybrid systems to determine whether charge transfer occurs between the polymers and ${\mathrm{TiO}}_{2}$ polymorphs upon photoexcitation. Furthermore, we evaluated whether the addition of the phenyl group alters the charge transfer mechanism, shifting it from a direct transfer (from the HOMO of the molecule to the conduction band of ${\mathrm{TiO}}_{2}$) to an indirect mechanism, where the electron transitions to the LUMO of the molecule before transferring to the ${\mathrm{TiO}}_{2}$ conduction band.

To synthesize ${\mathrm{TiO}}_{2}$ in the anatase phase, a hydrothermally assisted method using ethanol as a solvent was employed (see Section 2) [15]. However, the same procedure cannot be directly applied to produce the rutile phase. Usually, rutile-phase ${\mathrm{TiO}}_{2}$ requires very high temperatures (over 600 °C) for the conventional calcination techniques, and it requires the use of acidic alcohol-based solvents when performing hydrothermal treatment (generally above 200 °C). These methods are unsuitable for hybrid materials because the organic components, such as polymers, can break down at high temperatures and/or under extreme conditions. In our method, rutile ${\mathrm{TiO}}_{2}$ was obtained at only 60 °C, without using any organic solvents, surfactants, or harsh treatments. An alternative approach involves modifying the hydrothermal method used for anatase synthesis by replacing ethanol with water and using TiCl_4_ as the titanium precursor [60]. In previous studies, this method was followed by additional hydrothermal treatment to produce rutile ${\mathrm{TiO}}_{2}$ [60,61]. To address this, we extended the duration of the initial solution-based process and omitted the autoclave step. Titanium was hydrolyzed by OH groups in water and then slowly crystallized into ${\mathrm{TiO}}_{2}$. The resulting larger crystallite dimensions favored the formation of the rutile phase over the anatase phase [62,63]. TiCl_4_ reacts with water in a fast hydrolysis reaction, producing hydrated ${\mathrm{TiO}}_{2}$, and hydrochloric acid (HCl). The HCl makes the solution very acidic (pH below 2), favoring the formation of the rutile phase instead of anatase. In addition, the chloride ions in the solution help stabilize the rutile structure [64]. This makes our approach a simple, low-temperature, and eco-friendly method for making rutile ${\mathrm{TiO}}_{2}$, especially in combination with materials such as PhCN.

We produced a total of three sets of samples:

- Two PhCN/${\mathrm{TiO}}_{2}$ hybrids synthesized either with ethanol or water and thus in the anatase and rutile phases, respectively (PhCN/anatase and PhCN/rutile), to verify the charge transfer mechanism and assess the potential of the hybrid structure for visible, solar-driven applications;
- g-$C_{3}N_{4}$/${\mathrm{TiO}}_{2}$ synthesized with water and thus in the rutile phase (g-$C_{3}N_{4}$/rutile) to explore the specific role of the phenyl group.

**Figure 3:**
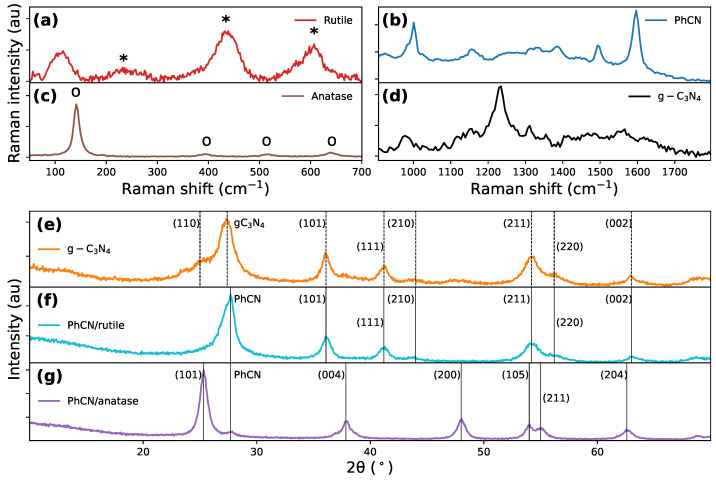
Raman spectra of (**a**) rutile, (**b**) PhCN, (**c**) anatase, and (**d**) g-$C_{3}N_{4}$. XRD patterns for (**e**) g-$C_{3}N_{4}$/rutile, (**f**) PhCN/rutile, and (**g**) PhCN/anatase. The positions of the ∗ (**a**) and *o* (**b**) peaks are reported in the text.

To assess the effectiveness of this modified approach, we synthesized pristine anatase and rutile phases by following both methods and analyzed their crystalline forms, ensuring the viability of the process before applying it to hybrid structures. The Raman spectra of ${\mathrm{TiO}}_{2}$ polymorphs (Figure 3a,c) revealed that for the sample synthesized via the hydrothermal method, the main peaks occur at 144, 398, 518, and 640$\;{\mathrm{cm}}^{-1}$, corresponding to the Eg (144 and 398), A1g, and Eg vibrational modes of anatase crystallites, respectively, (Figure 3c). Conversely, the Raman spectrum of the water-assisted-synthesized sample revealed peaks at 210 $\;{\mathrm{cm}}^{-1}$ (B1g), 448 $\;{\mathrm{cm}}^{-1}$ (Eg), and 613 $\;{\mathrm{cm}}^{-1}$ (A1g), characteristic of the rutile phase (Figure 3a) [63,65], proving the efficacy of the alternative synthesis approach. In the case of the hybrid compounds, exciting the sample with an infrared laser (1064 nm) allowed a clear observation of the leading bands of the two polymorphs, overcoming the interference caused by the luminescence of heptazine (Figure 3b,d). This confirmed the effective adhesion of the organic molecules on ${\mathrm{TiO}}_{2}$ surfaces.

Regarding the structural characterization, the XRD pattern of the hydrothermal compound (PhCN/anatase) reveals all the characteristic peaks of the anatase phase, along with a minor peak at 25°, corresponding to the (101) plane of anatase ${\mathrm{TiO}}_{2}$ (Figure 3g). The XRD patterns of the water-synthesized samples (g-$C_{3}N_{4}$/rutile and PhCN/rutile) exhibit similar features, with a prominent peak at approximately 27° (Figure 3e,f), corresponding to the (001) reflection (slightly varying between 27.5° and 27.7° due to interplanar distance changes), and a broader peak at around 15°, generally assigned to the (210) reflection from the separation distance of heptazine chains [10] but not clearly distinguishable from our spectra due to the large background coming from the ${\mathrm{TiO}}_{2}$ contribution. These organic peaks overlap with the dominant peaks of the rutile phase, with its main peak also appearing at 27°. In the g-$C_{3}N_{4}$/rutile compound, the presence of a small amount of anatase is indicated by a shoulder at 25° (Figure 3e). These patterns provide clear evidence of the adhesion of the organic molecules on both ${\mathrm{TiO}}_{2}$ phases. The detection of organic-specific peaks in conjunction with ${\mathrm{TiO}}_{2}$ diffraction patterns highlights the integration of the two components. While XRD and Raman data confirm phase purity and chemical bonding, we acknowledge that high-resolution morphological data (SEM, TEM, and HRTEM) would further elucidate the particle size distribution, interface structure, and dispersion of ${\mathrm{TiO}}_{2}$ on the PhCN matrix.

### 3.4. Experimental Results: Absorption and Emission Spectra

Optical characterization provides valuable insights into the structural differences and interactions between the organic and inorganic parts. ${\mathrm{TiO}}_{2}$, in both its anatase and rutile phases, strongly absorbs light in the UV range, with a sharp increase in absorption for wavelengths shorter than 410 nm, corresponding to its bandgap (∼3.2 eV for anatase and ∼3.0 eV for rutile). In contrast, g-$C_{3}N_{4}$ exhibits optical absorption below 450 nm (bandgap ∼2.7 eV).

**Figure 4:**
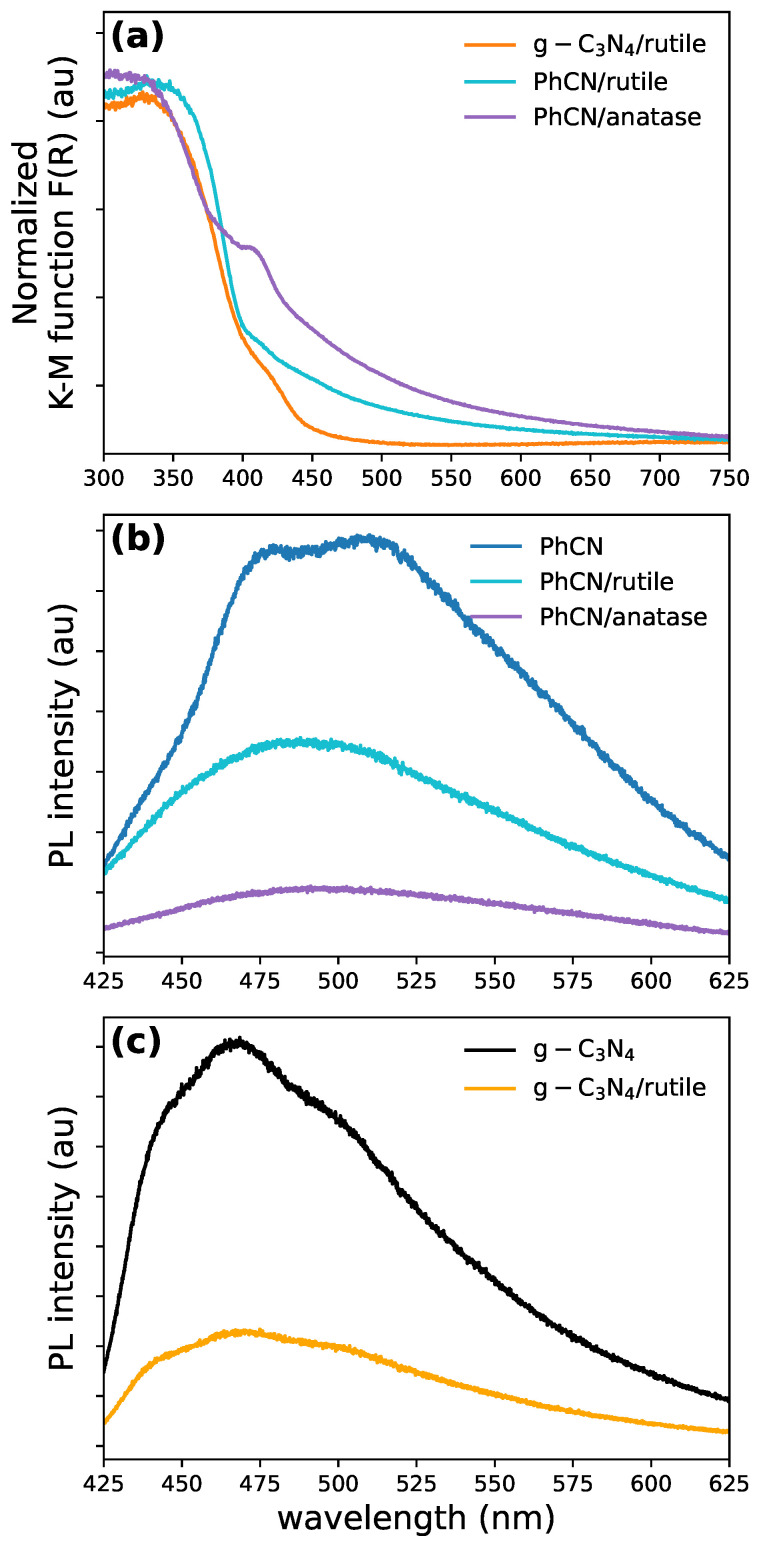
(**a**) Absorption spectra of g-$C_{3}N_{4}$–PhCN hybrid systems. Comparison of emission spectra for (**b**) PhCN, PhCN/rutile, PhCN/anatase, and (**c**) g-$C_{3}N_{4}$ and g-$C_{3}N_{4}$/rutile.

At the same time, absorption by PhCN extends across the entire visible range due to the presence of phenyl groups in its structure (Figure S3). This aligns with the previous theoretical predictions, where we showed that adding a phenyl group would result in an overall decrease in the HOMO–LUMO gap, leading to a redshift in the absorption spectrum. The composites with organic structures show an expanded working range between 400 and 600 nm (Figure 4a). However, the high-energy portion of the spectra shows minimal differences between anatase and rutile phases. As anticipated above, the two hybrid systems exhibit distinct absorption mechanisms. In the PhCN/rutile system, there is photoinduced electron transfer (Figure 2b), where excited electrons in the LUMO of PhCN are transferred to the conduction band of ${\mathrm{TiO}}_{2}$. Conversely, the g-$C_{3}N_{4}$/rutile complex operates through direct optical electron transfer (Figure 2a), where photons promote electrons directly from the HOMO ground state of g-$C_{3}N_{4}$ to the conduction band of ${\mathrm{TiO}}_{2}$ [1,15]. This direct transfer mechanism results in a redshifted optical absorption compared to the simple sum of the absorption features of the individual components, regardless of the ${\mathrm{TiO}}_{2}$ polymorph used (see Figure 4a). The observed behavior confirms our calculations, where we showed that adding a phenyl group would result in an increase in $\Delta^{\prime }$ (see Table 4), indicating that an indirect electron transfer mechanism in the case of PhCN/rutile is favorable. At the same time, it might follow a direct pathway in g-$C_{3}N_{4}$/rutile. The luminescence of the hybrid samples revealed the difference induced by the phenyl group. The excitation/emission characteristics of the photoluminescence of the five samples are reported in Figure 4b,c. While the inorganic parts do not seem to contribute significantly, the presence of the phenyl groups in the heptazine mesh generates a redshift of the emission. In carbon nitride systems, photoluminescence (PL) arises from recombination between the σ* and lone pair (LP), π* and LP, and π* and π energy levels [51,66]. These recombinations create a broad emission spectrum centered around 530 nm for PhCN (Figure 4b). In contrast, in g-$C_{3}N_{4}$, the emission is dominated by transitions from σ* to LP levels, generating a blueshifted spectrum (Figure 4c) and, most importantly, a strong reduction in PL efficiency due to competitive thermal recombination from the π* levels.

### 3.5. Experimental Results: Photocatalytic Efficiency

**Figure 5:**
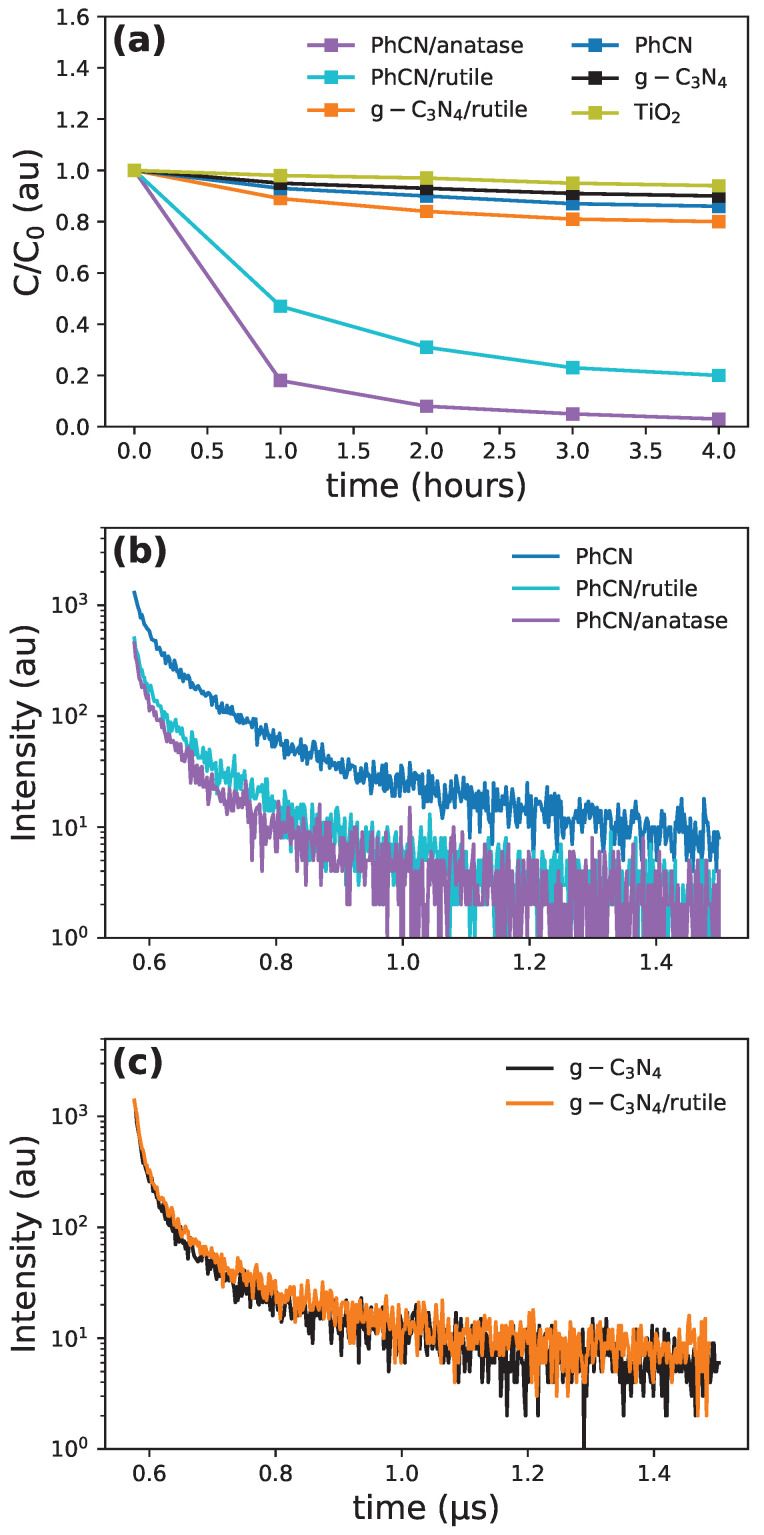
(**a**) Photocatalytic efficiency for RhB degradation. PL decay profiles for (**b**) PhCN and PhCN hybrids and for (**c**) g-$C_{3}N_{4}$ and g-$C_{3}N_{4}$/rutile structures.

The formation of an active heterostructure can be directly tested by photocatalytic properties (Figure 5a). Although it has already been proved that PhCN with anatase forms an efficient photocatalyst activated by visible light, the use of g-$C_{3}N_{4}$ as the organic part in the hybrid compound with ${\mathrm{TiO}}_{2}$ reduces the activity if the incident light has a wavelength higher than 450 nm [15].

In the PhCN/rutile sample, the photocatalytic activity is slightly reduced with respect to the anatase sample but is still noteworthy, indicating an effective charge transfer from PhCN to rutile. In contrast, the absence of the phenyl group in the organic part strongly reduces the connection between g-$C_{3}N_{4}$ and the rutile structure, as indicated by the lower photocatalytic activity. Time-resolved photoluminescence (TRPL) measurements help in quantifying these aspects: the information from TRPL data is connected to the probability of recombination from the excited states via the relation

(2) $$\gamma_{\mathrm{tot}}=\gamma_{\mathrm{rad}}+\gamma_{\mathrm{NR}}=\frac{1}{\tau_{\mathrm{rad}}}+\frac{1}{\tau_{\mathrm{NR}}}=\frac{1}{\tau_{\mathrm{tot}}}$$

where $\gamma_{\mathrm{tot}}$ is the recombination probability from the excited state, with $\gamma_{\mathrm{rad}}$ and $\gamma_{\mathrm{NR}}$ as the probabilities of radiative and non-radiative recombination, respectively. $\tau_{\mathrm{rad}}$ and $\tau_{\mathrm{NR}}$ are the relative lifetimes of the radiative, and non-radiative paths and $\tau_{\mathrm{tot}}$, the overall decay time, is obtained by fitting the measured signal (see Figure 5b,c); therefore, a decrease in the experimental luminescence decays is a clear indication of the formation of non-radiative pathways. In Table 5, we report the weighted average of the time decay constant, together with the efficiency of the non-radiative charge transfer mechanism compared to PhCN, calculated as follows:

(3) $$\eta_{\mathrm{CT}}=1-\tau_{\mathrm{hybrid}}/t_{\mathrm{PhCN}}$$

While it seems that there is almost no charge transfer from g-$C_{3}N_{4}$ (which aligns with the low absorption of the polymer in the visible range), the charge transfer efficiency for the PhCN hybrids is about 40% for both the ${\mathrm{TiO}}_{2}$ phases. The increased efficiency in the anatase-based hybrid system is most probably related to the more active role of anatase with respect to rutile in the photocatalytic process. However, the high efficiency of photocatalysis for the rutile heterostructure opens new possibilities for environmentally friendly and cost-efficient solutions.

## 4. Conclusions

In this study, we investigated the photocatalytic properties of phenyl-modified carbon nitride in combination with the rutile phase of ${\mathrm{TiO}}_{2}$ using a combined computational and experimental approach. DFTB calculations proved the effective interaction of PhCN with rutile, favorable band alignment, and enhanced charge transfer efficiency. These results indicate efficient charge separation and reduced recombination, supporting the potential of PhCN/rutile for photocatalytic applications. Furthermore, phenyl functionalization improves visible light absorption by reducing the energy gap between the highest occupied molecular orbital (HOMO) and the lowest unoccupied molecular orbital (LUMO). Raman spectroscopy, XRD, and photocatalytic degradation measurements confirmed the computational predictions. These analyses showed the successful integration of PhCN onto both anatase and rutile ${\mathrm{TiO}}_{2}$, demonstrating effective charge transfer interactions. Photoluminescence and time-resolved spectroscopy revealed approximately 40% charge transfer efficiency in both ${\mathrm{TiO}}_{2}$ phases, aligning with computational findings. Additionally, photocatalytic experiments confirmed significant activity under visible light, supporting the practical viability of PhCN/rutile as a green and sustainable photocatalyst, emphasizing its role as an effective alternative to anatase-based systems.

## Figures and Tables

**Table 1:** Cell parameters of bulk rutile and anatase calculated by both the DFT and DFTB methods. The third column represents the relative deviation of the DFTB value compared to the full ab initio one.

		DFT (Å)	DFTB (Å)	
Rutile	a	4.648	4.619	0.63%
	c	2.971	2.991	−0.68%
Anatase	a	3.805	3.758	1.26%
	c	9.747	9.605	1.45%

**Table 2:** DFTB-calculated formation energies for the (100) and (110) facets of both anatase and rutile. The comparison is made with the range of values available in the literature, although they were obtained with different functionals and pseudopotentials.

		Anatase (J/m^2^)	Rutile (J/m^2^)
DFTB	100	0.85	1.09
	110	1.39	0.88
Different XC [45]	100	0.53–0.90	0.67–0.77
PBE-D4+U [46]	110	0.95–1.32	0.48–0.54

**Table 3:** Absorption energies of the molecules investigated herein.

$E_{\mathrm{abs}}$ (eV)		Triazine	Ph-Triazine	Heptazine	Ph-Heptazine
Anatase	100	−0.59	−0.76	−0.64	−0.63
	110	−0.82	−0.99	−0.96	−1.21
Rutile	100	−0.27	−0.18	−0.50	−0.42
	110	−0.54	−0.86	−0.58	−0.95

**Table 4:** Energy differences between the electronic levels of the molecule and the ${\mathrm{TiO}}_{2}$ substrate for all the cases investigated in this work. All energies are reported in eV. A complete collection of the energy levels for the systems investigated here is reported in Table S1.

Substrate		Molecule	Δ	$\Delta^{\prime }$
100	Anatase	Triazine	2.34	1.89
		Ph-triazine	1.05	2.16
	Rutile	Triazine	4.34	0.75
		Ph-triazine	2.36	1.07
110	Anatase	Triazine	2.99	1.66
		Ph-triazine	1.11	2.23
	Rutile	Triazine	2.99	1.66
		Ph-triazine	1.60	1.23
100	Anatase	Heptazine	1.92	1.44
		Ph-heptazine	0.96	1.63
	Rutile	Heptazine	2.94	0.57
		Ph-heptazine	1.81	0.76
110	Anatase	Heptazine	1.62	1.68
		Ph-heptazine	0.61	1.87
	Rutile	Heptazine	2.90	0.60
		Ph-heptazine	1.36	0.97

**Table 5:** Weighted averaged of time decay obtained through the fit shown in Figure 5b,c, together with the charge transfer efficiency $\eta_{\mathrm{CT}}$ calculated via Equation (3).

Sample	$\tau_{\mathrm{avg}}$ (ns)	$\eta_{\mathrm{CT}}$
g-$C_{3}N_{4}$	9.4	
g-$C_{3}N_{4}$/water	9.3	0.007
PhCN	19.7	
PhCN/water	12.2	0.380
PhCN/ethanol	11.5	0.417

## Data Availability

The simulation cells for all the structures investigated in this paper and the experimental data are available at http://github.com/rdettori/PhCN_TiO2 (accessed on 3 April 2025).

## Supplementary Materials

The following supporting information can be downloaded at: https://www.mdpi.com/article/10.3390/polym17101331/s1.
