# Supplementary material for: Sustainable Photocatalysis with Phenyl-Modified g-C3N4/TiO2 Polymer Hybrids: A Combined Computational and Experimental Investigation
Source: Polymers (Basel). 2025 May 14;17(10):1331. doi: 10.3390/polym17101331 (PMC12114864; doi:10.3390/polym17101331)
Supplement: Supplementary file 1 [file polymers-17-01331-s001.zip › polymers-3597716-supplementary.pdf]

Supporting Information:
Phenyl-modified $g-C_3N_4/TiO_2$ hybrids: a combined computational and experimental investigation for sustainable photocatalysis

Riccardo Dettori*, Sahar Aghapour Ghourichay, Stefania Porcu, Claudio Melis, Luciano Colombo, and Pier Carlo Ricci
[1] *Department of Physics, University of Cagliari, Monserrato, CA, 09042 Italy*
(*riccardo.dettori@dsf.unica.it)

I. SIMULATION DETAILS

A. Sample generation and assessment of simulation parameters

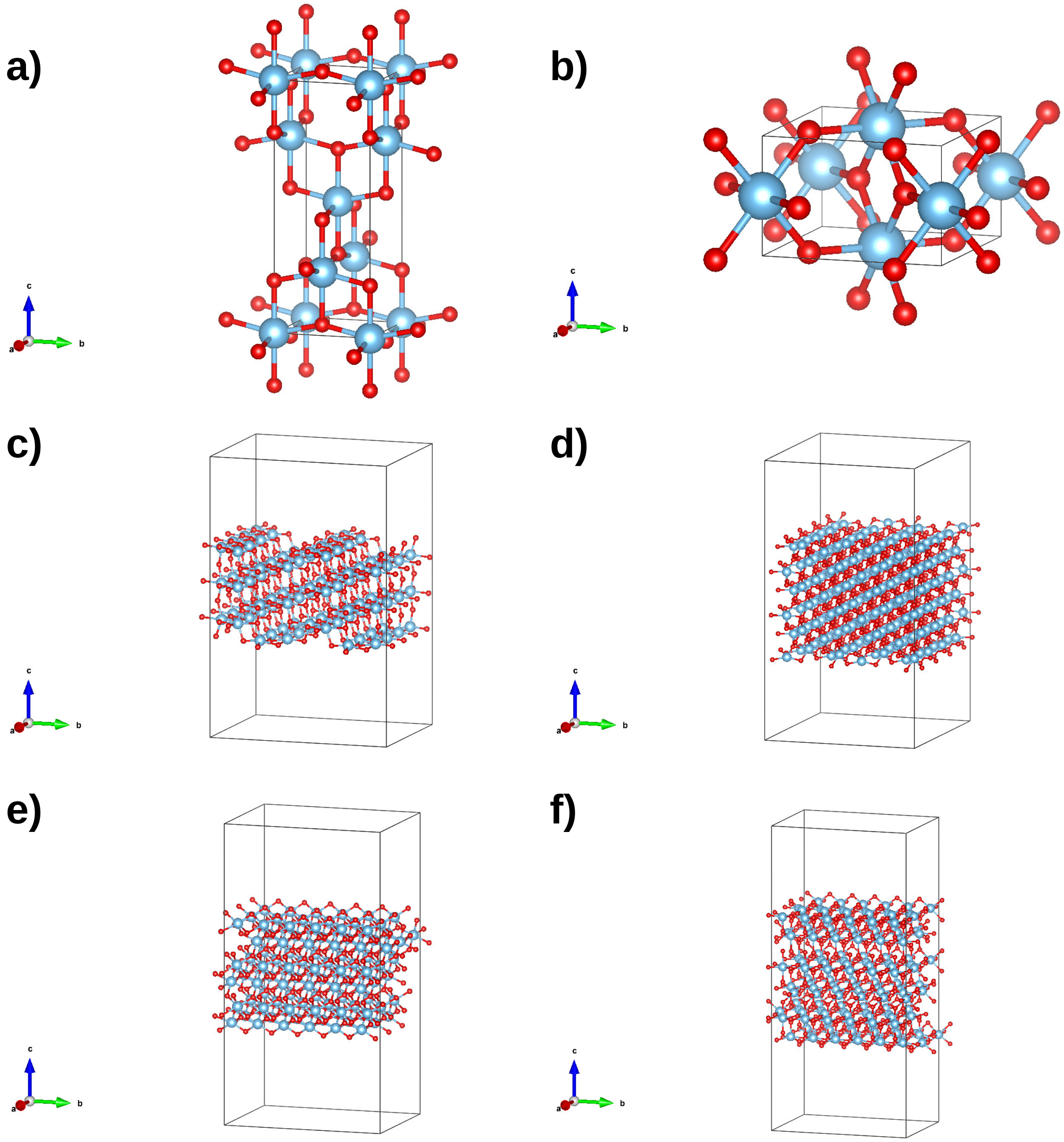

FIG. 1. (a) Bulk structure of anatase, (b) bulk structure of rutile, (c) (100) oriented facet of anatase, (d) (100) oriented facet of rutile, (e) (110) oriented facet of anatase, and (f) (110) oriented facet of rutile.

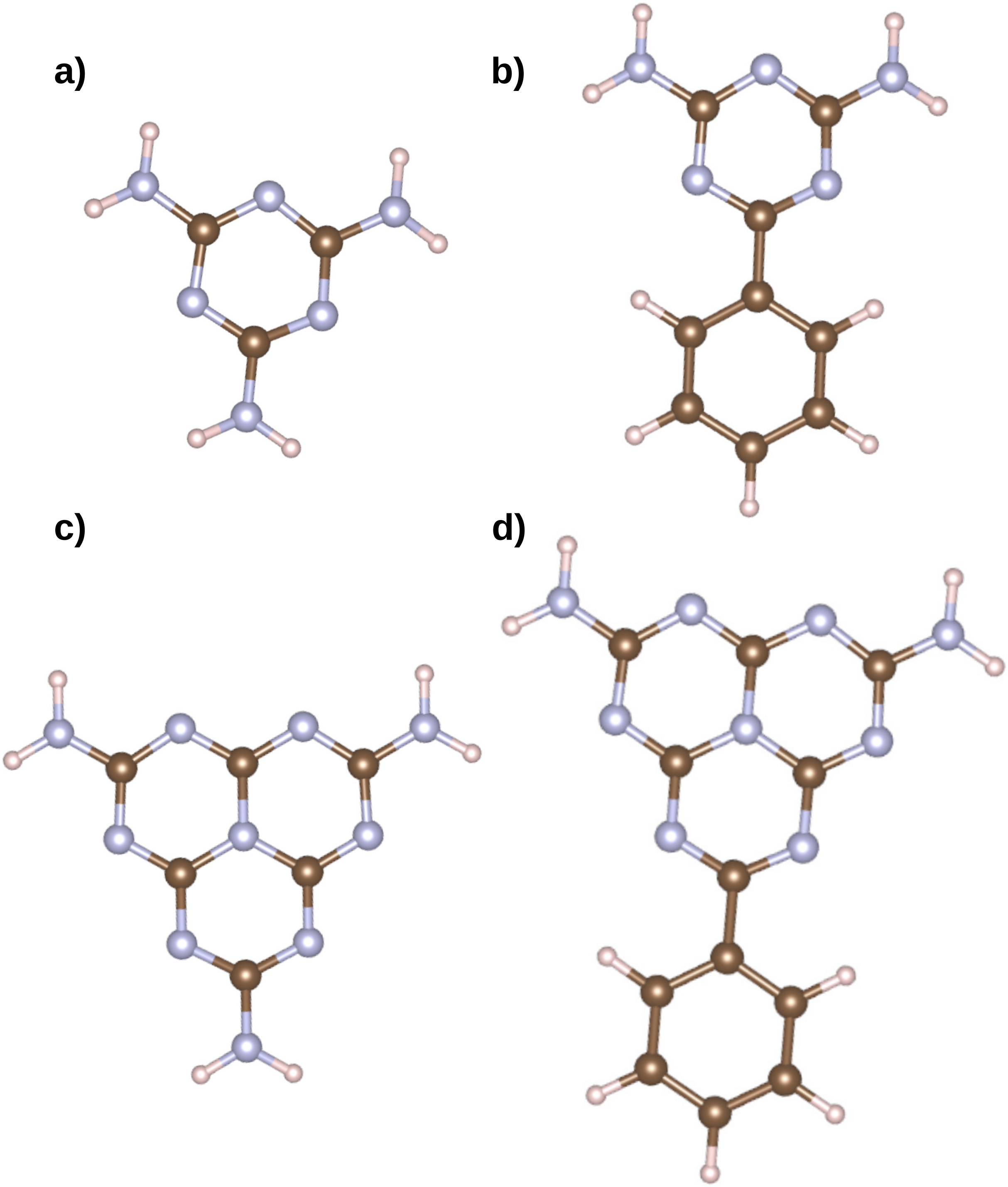

FIG. 2. Atomistic representation of the molecules considered in this work. (a) Triazine, (b) Ph-triazine, (c) heptazine, (d) Ph-triazine.

B. Triazine and Heptazine samples

II. RESULTS

A. Density of states and band alignment

Here is the complete collection of the DOS for the 16 structures investigated and the construction procedure for the band alignment, along with the computed Δ and Δ'. Energy levels and energy differences are reported in Tab. I.

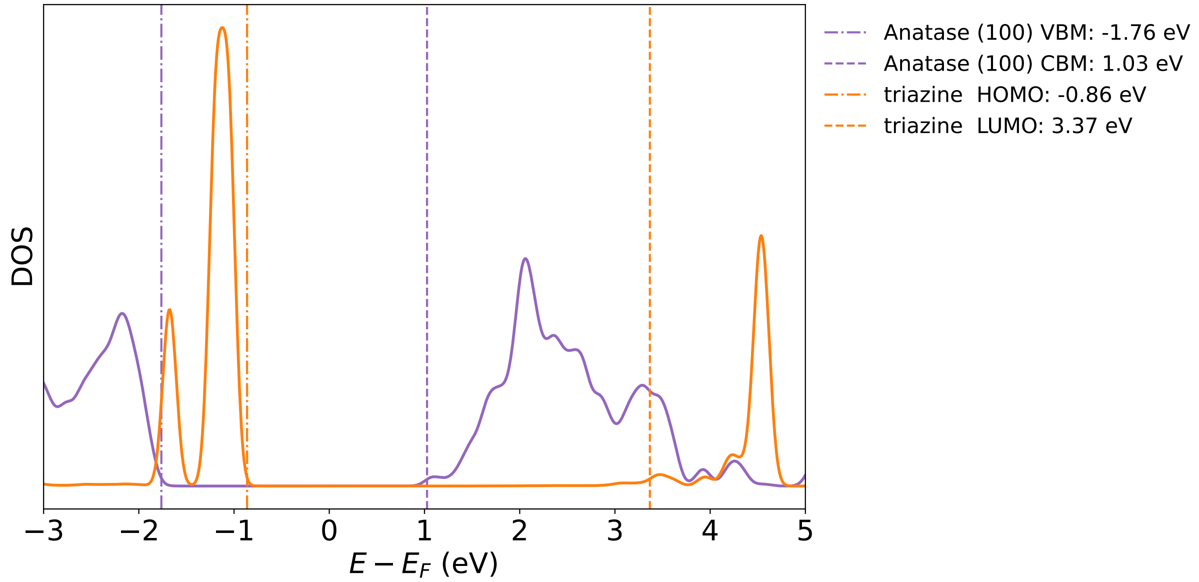

TABLE I. Energy levels of the molecule and the TiO_2 substrate for all the cases investigated in this work. All energies are reported in eV.

Substrate		Molecule	VBM	CBM	HOMO	LUMO	Δ	Δ'
100	Anatase	triazine	-1.76	1.03	-0.86	3.37	2.34	1.89
		Ph-triazine	-1.62	1.15	-1.01	2.20	1.05	2.16
	Rutile	triazine	-2.18	0.41	-0.34	4.75	4.34	0.75
		Ph-triazine	-2.02	0.57	-0.50	2.93	2.36	1.07
110	Anatase	triazine	-1.90	0.95	-0.71	3.94	2.99	1.66
		Ph-triazine	-1.60	1.23	-1.00	2.34	1.11	2.23
	Rutile	triazine	-1.90	0.95	-0.71	3.94	2.99	1.66
		Ph-triazine	-1.71	0.68	-0.55	2.28	1.60	1.23
100	Anatase	heptazine	-2.00	0.79	-0.65	2.71	1.92	1.44
		Ph-heptazine	-1.90	0.88	-0.75	1.84	0.96	1.63
	Rutile	heptazine	-2.28	0.32	-0.25	3.26	2.94	0.57
		Ph-heptazine	-2.19	0.40	-0.36	2.21	1.81	0.76
110	Anatase	heptazine	-1.90	0.95	-0.73	2.57	1.62	1.68
		Ph-heptazine	-1.79	1.04	-0.83	1.65	0.61	1.87
	Rutile	heptazine	-2.02	0.36	-0.24	3.26	2.90	0.60
		Ph-heptazine	-1.83	0.54	-0.43	1.90	1.36	0.97

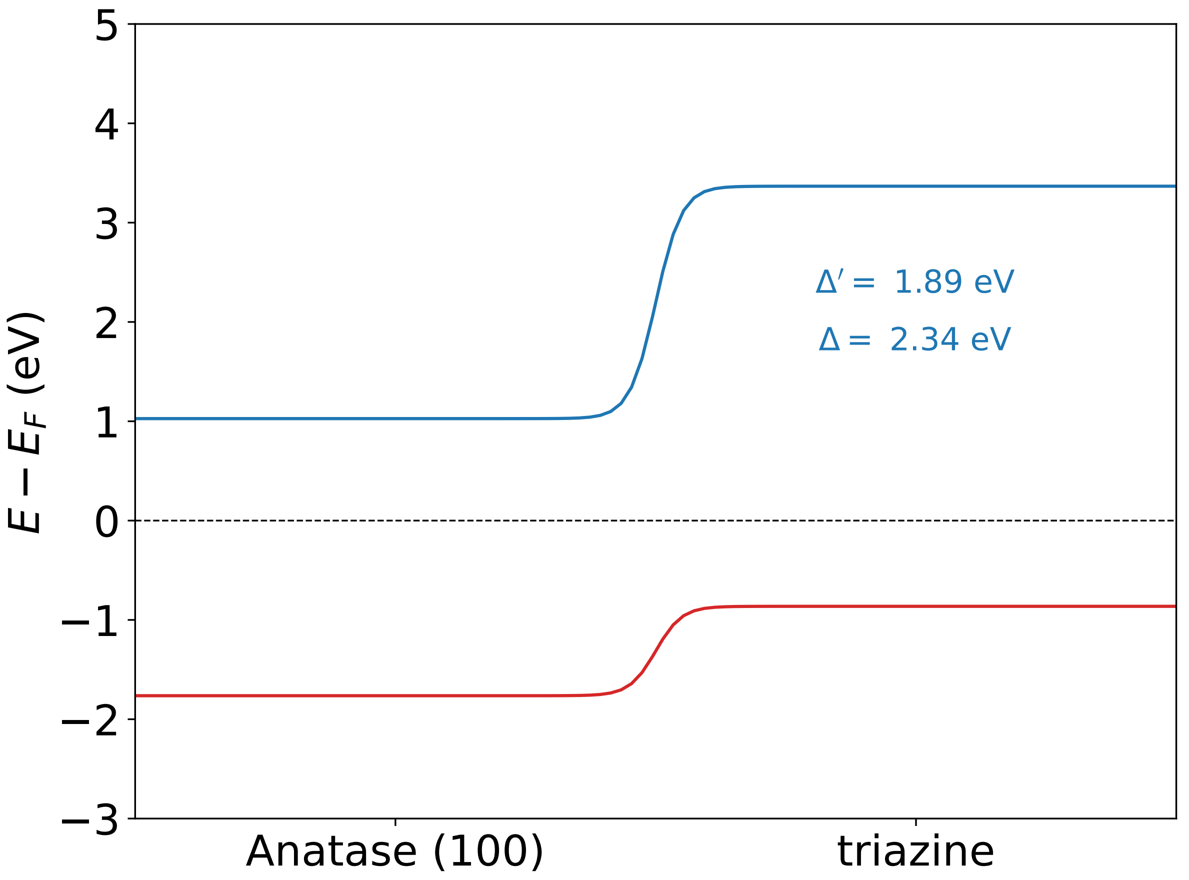
5
4
3
2
1
0
−1
−2
−3
E − E_F (eV)
Δ′ = 1.89 eV
Δ = 2.34 eV
Anatase (100)
triazine

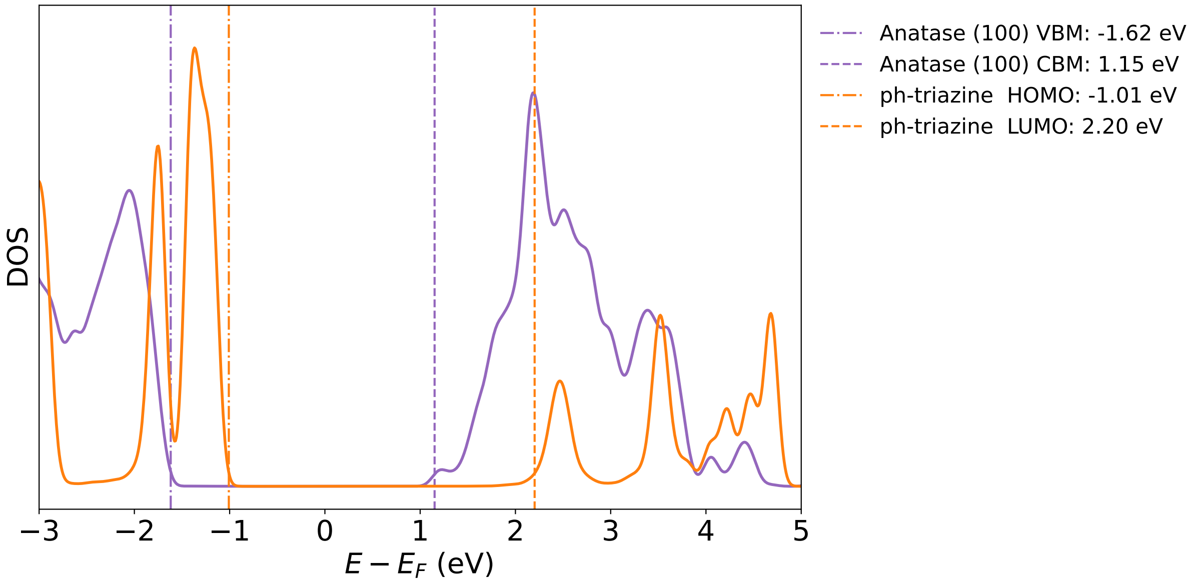
DOS
−3
−2
−1
0
1
2
3
4
5
E − E_F (eV)
Anatase (100) VBM: -1.62 eV
Anatase (100) CBM: 1.15 eV
ph-triazine HOMO: -1.01 eV
ph-triazine LUMO: 2.20 eV

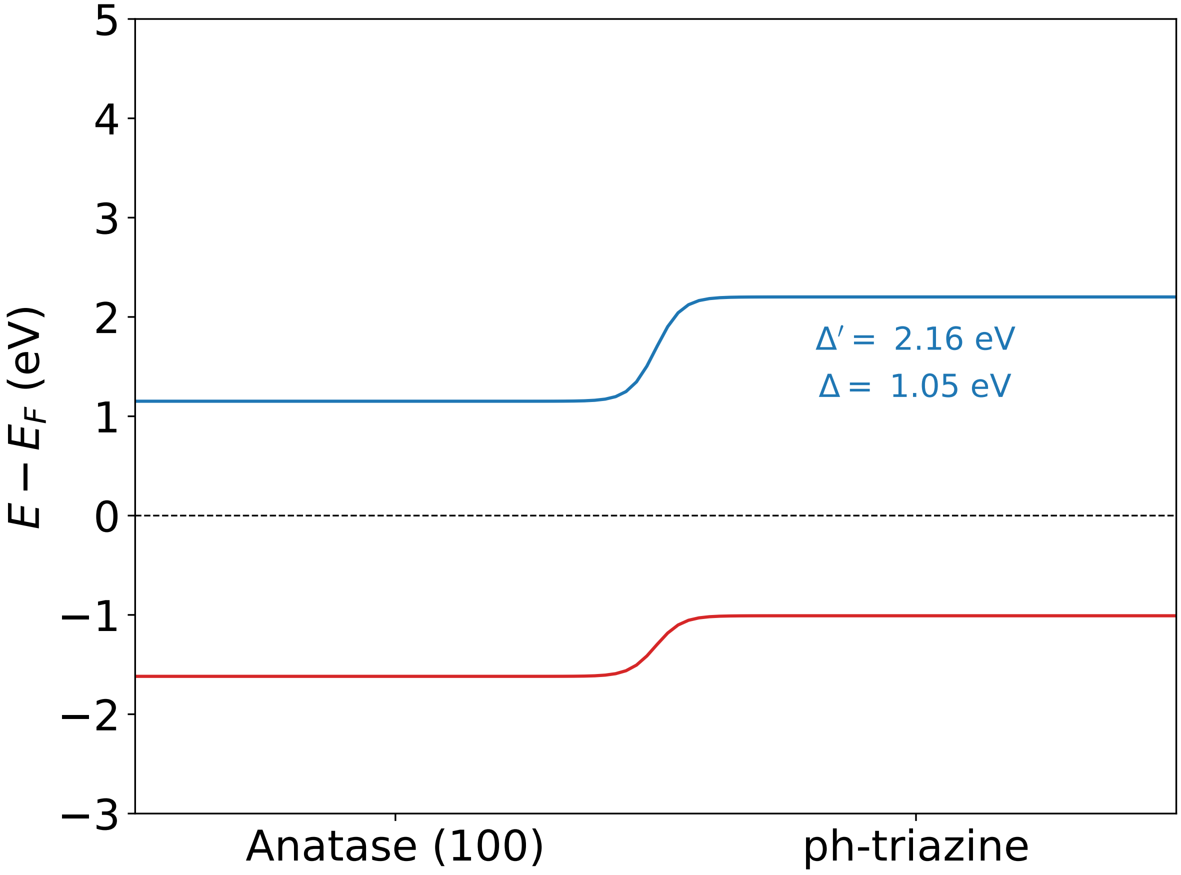

5
4
3
2
1
0
−1
−2
−3
$E - E_F$ (eV)
Δ′ = 2.16 eV
Δ = 1.05 eV
Anatase (100)
ph-triazine

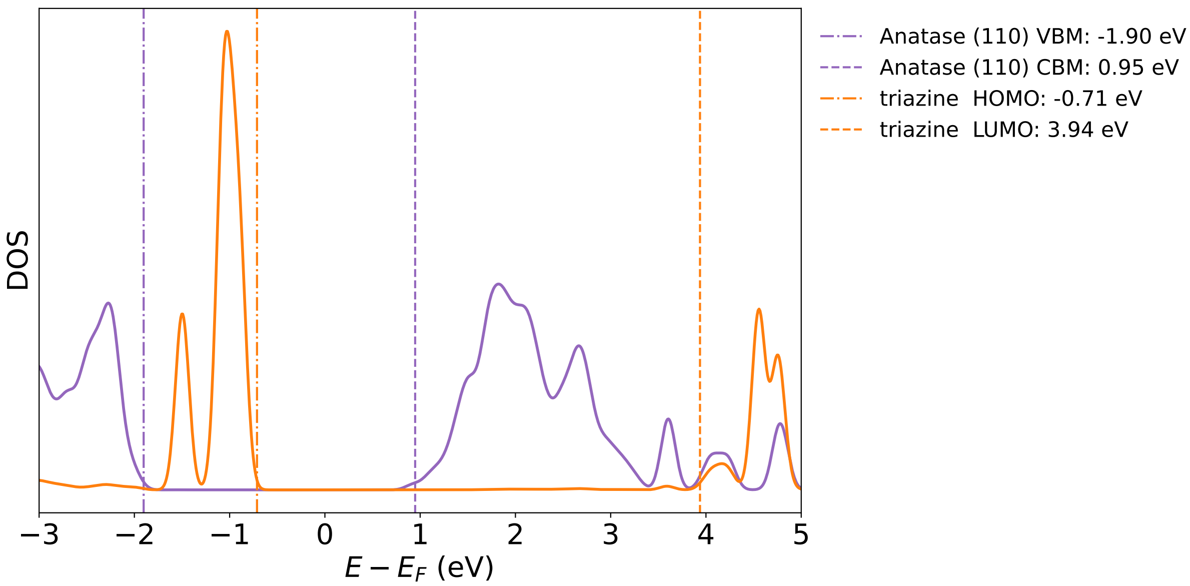

Anatase (110) VBM: -1.90 eV
Anatase (110) CBM: 0.95 eV
triazine HOMO: -0.71 eV
triazine LUMO: 3.94 eV
DOS
−3
−2
−1
0
1
2
3
4
5
$E - E_F$ (eV)

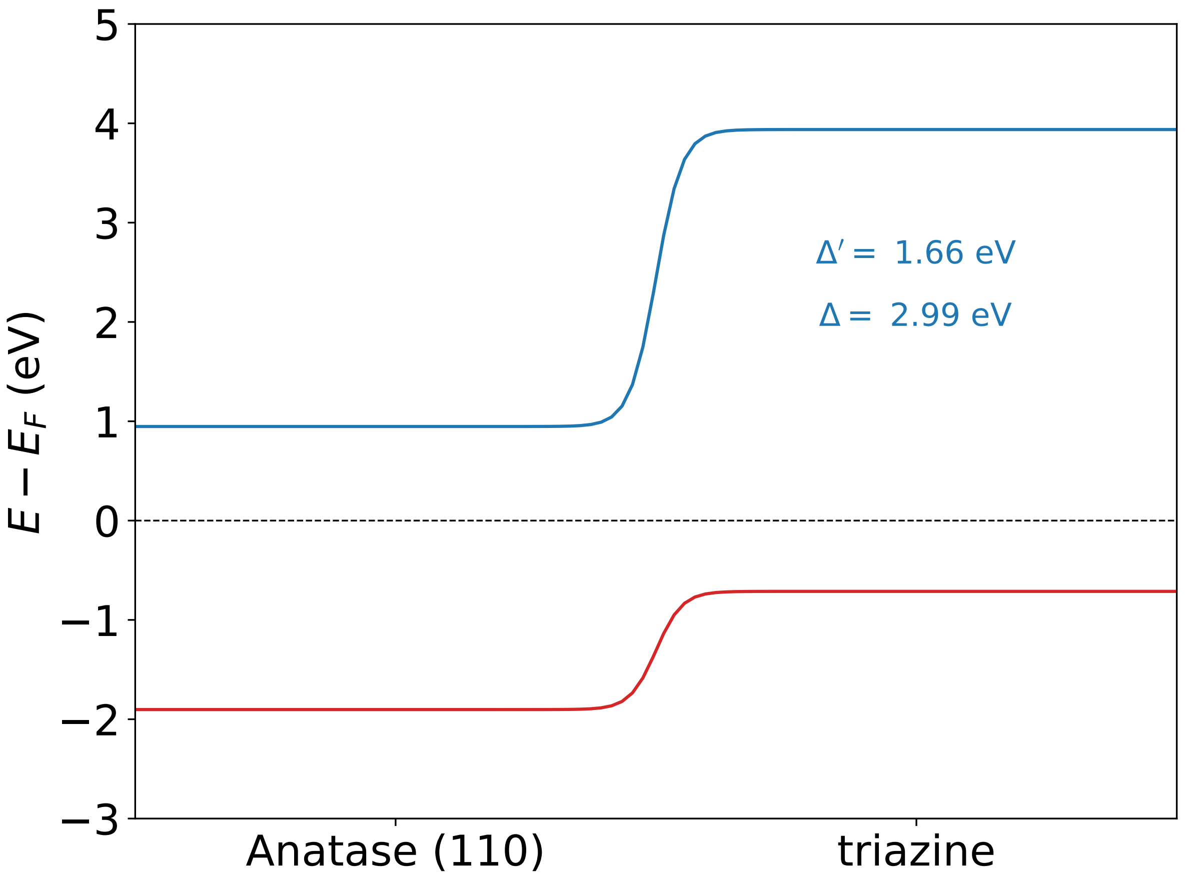
5
4
3
2
1
0
−1
−2
−3
$E - E_F$ (eV)
Δ′ = 1.66 eV
Δ = 2.99 eV
Anatase (110)
triazine

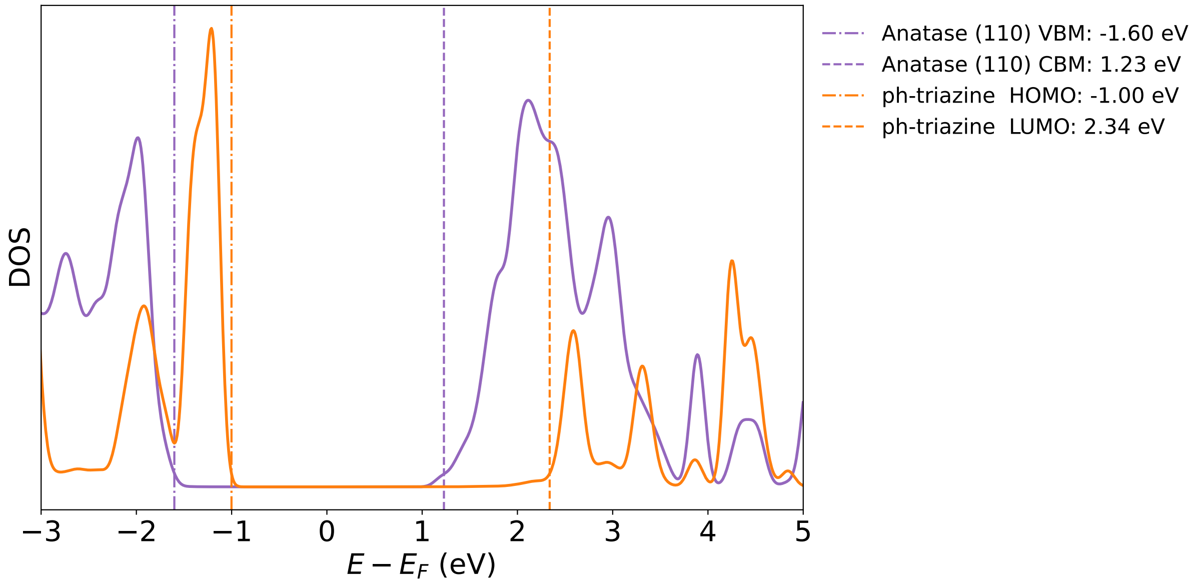
Anatase (110) VBM: -1.60 eV
Anatase (110) CBM: 1.23 eV
ph-triazine HOMO: -1.00 eV
ph-triazine LUMO: 2.34 eV
DOS
−3
−2
−1
0
1
2
3
4
5
$E - E_F$ (eV)

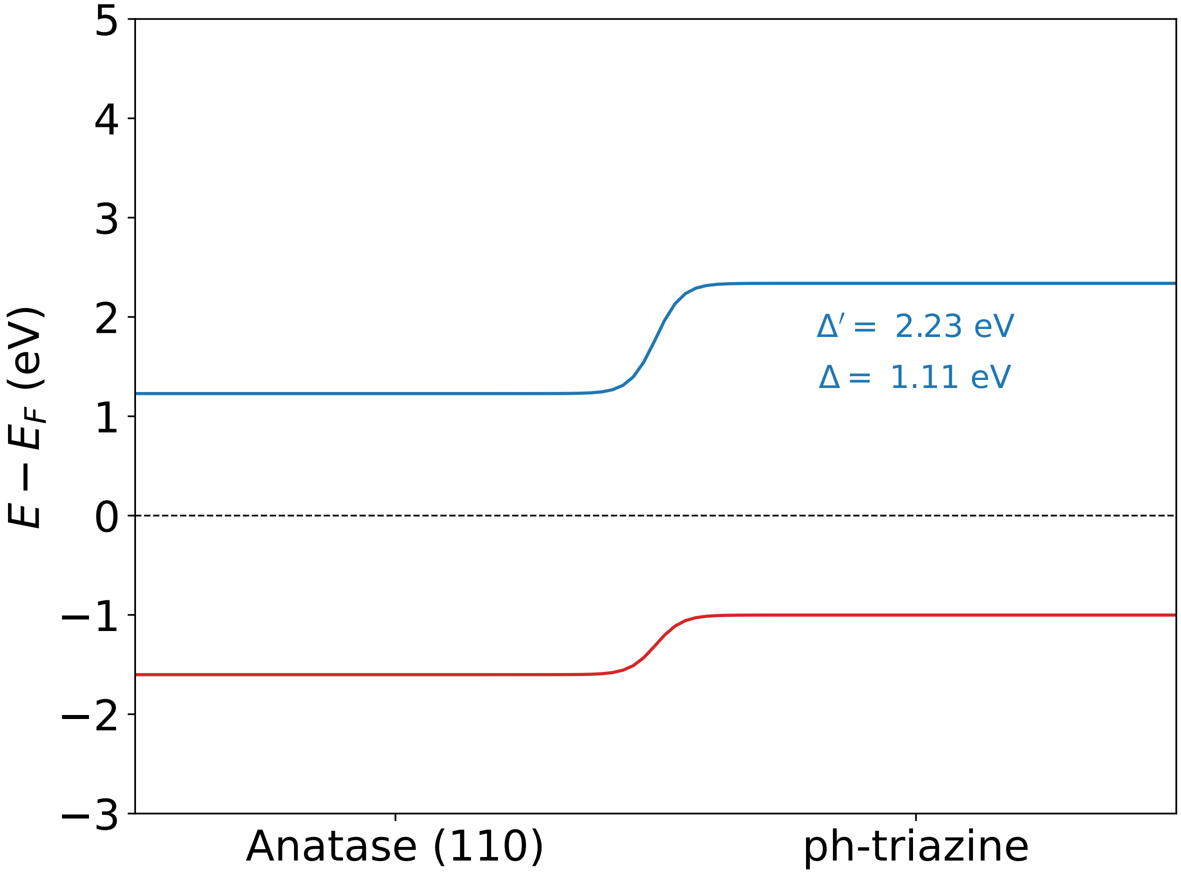
5
4
3
2
1
0
−1
−2
−3
$E - E_F$ (eV)
Δ′ = 2.23 eV
Δ = 1.11 eV
Anatase (110)
ph-triazine

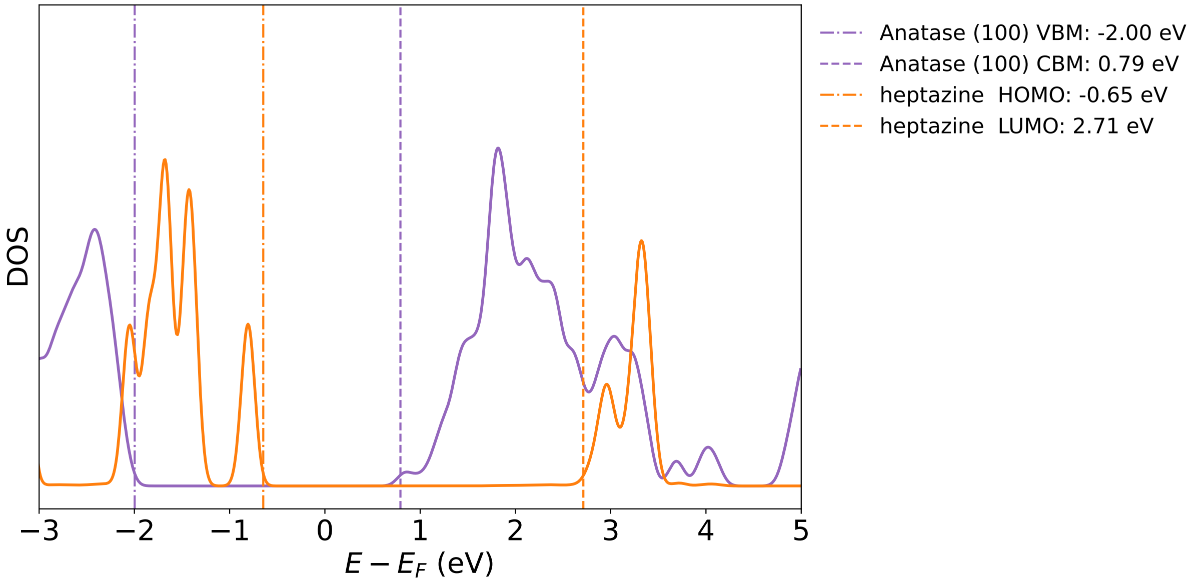
Anatase (100) VBM: -2.00 eV
Anatase (100) CBM: 0.79 eV
heptazine HOMO: -0.65 eV
heptazine LUMO: 2.71 eV
DOS
−3
−2
−1
0
1
2
3
4
5
$E - E_F$ (eV)

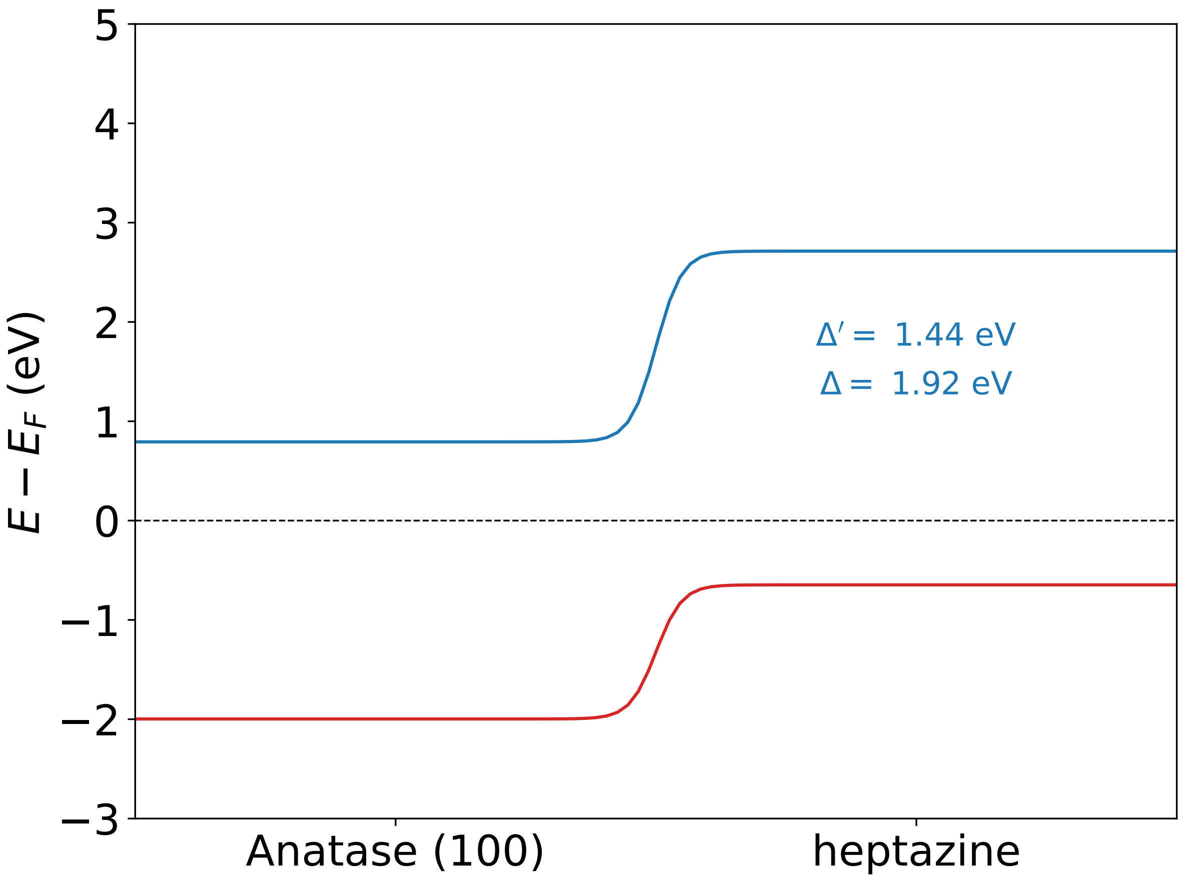

5
4
3
2
1
0
−1
−2
−3
$E - E_F$ (eV)
$\Delta' =$ 1.44 eV
$\Delta =$ 1.92 eV
Anatase (100)
heptazine

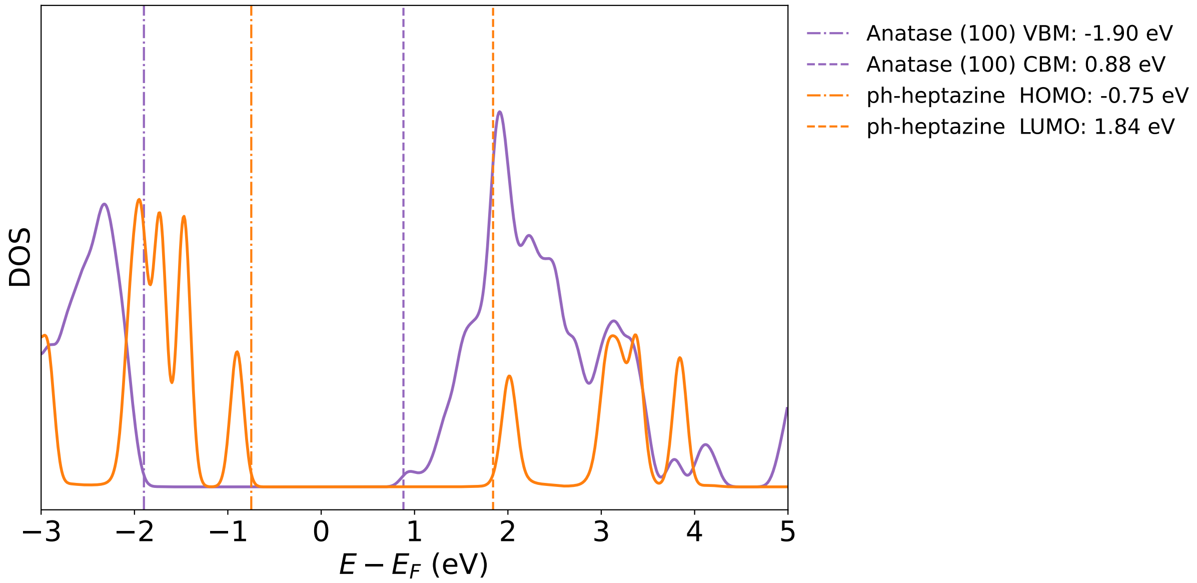

DOS
−3
−2
−1
0
1
2
3
4
5
$E - E_F$ (eV)
Anatase (100) VBM: -1.90 eV
Anatase (100) CBM: 0.88 eV
ph-heptazine HOMO: -0.75 eV
ph-heptazine LUMO: 1.84 eV

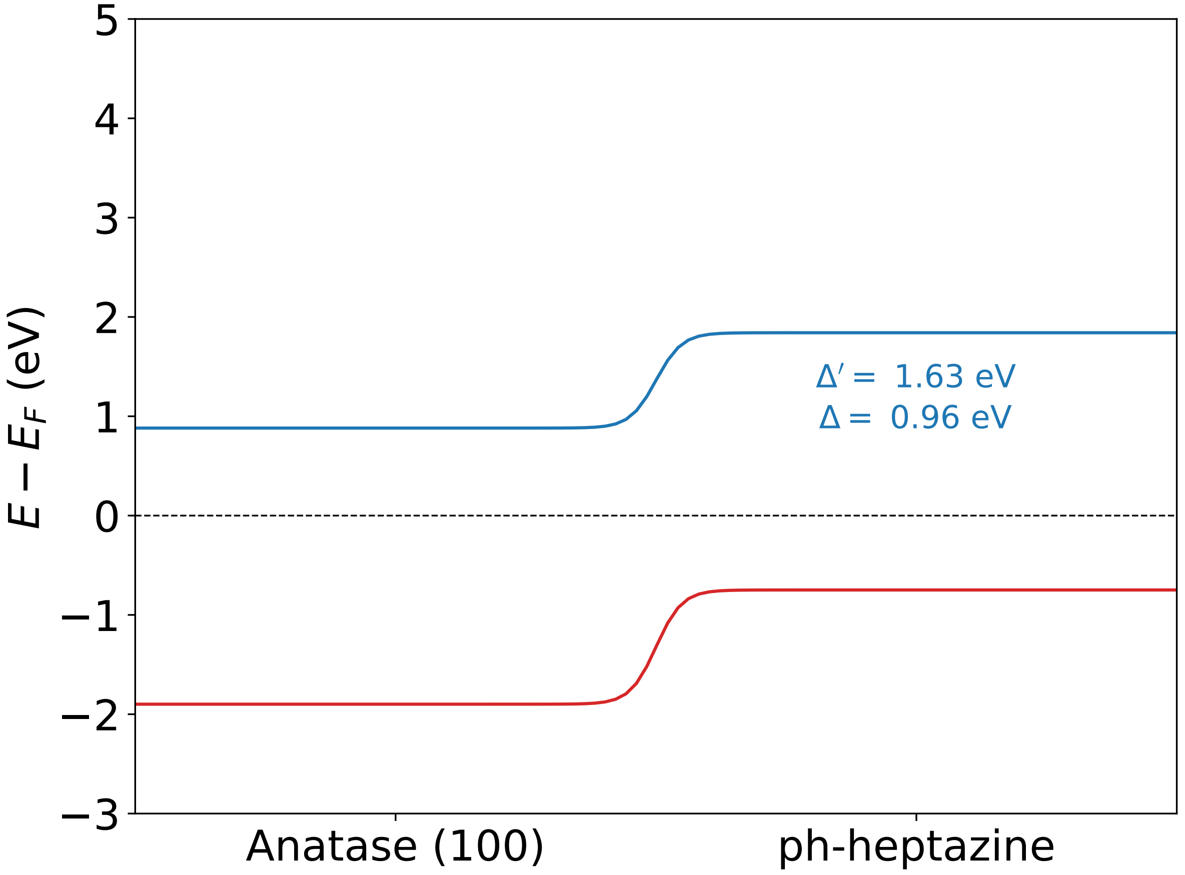

5
4
3
2
1
0
−1
−2
−3
$E - E_F$ (eV)
$\Delta' =$ 1.63 eV
$\Delta =$ 0.96 eV
Anatase (100)
ph-heptazine

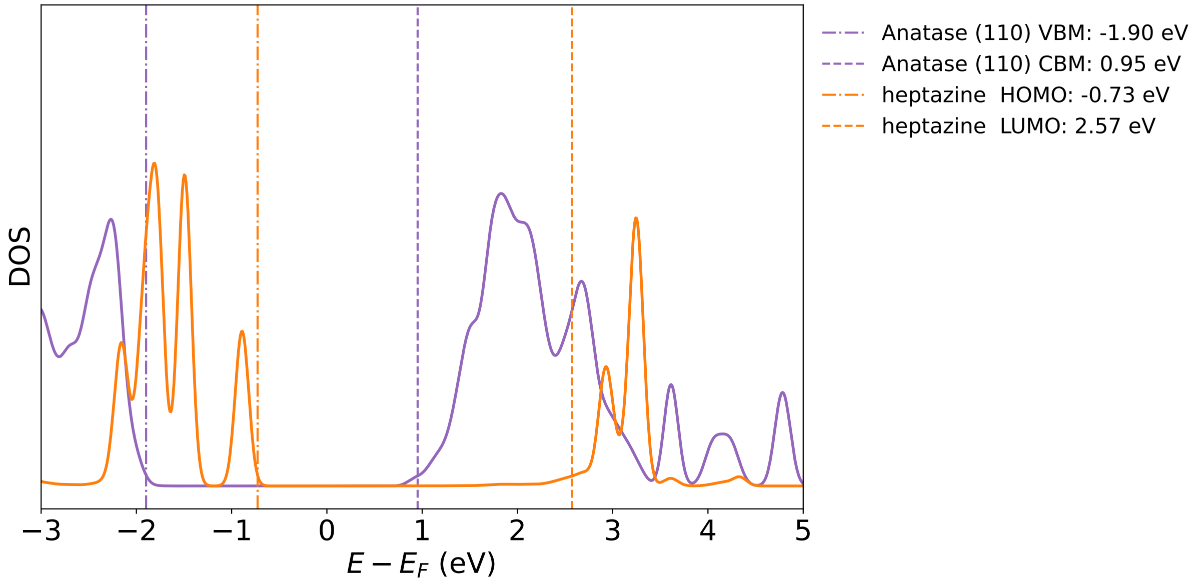

DOS
−3
−2
−1
0
1
2
3
4
5
$E - E_F$ (eV)
Anatase (110) VBM: -1.90 eV
Anatase (110) CBM: 0.95 eV
heptazine HOMO: -0.73 eV
heptazine LUMO: 2.57 eV

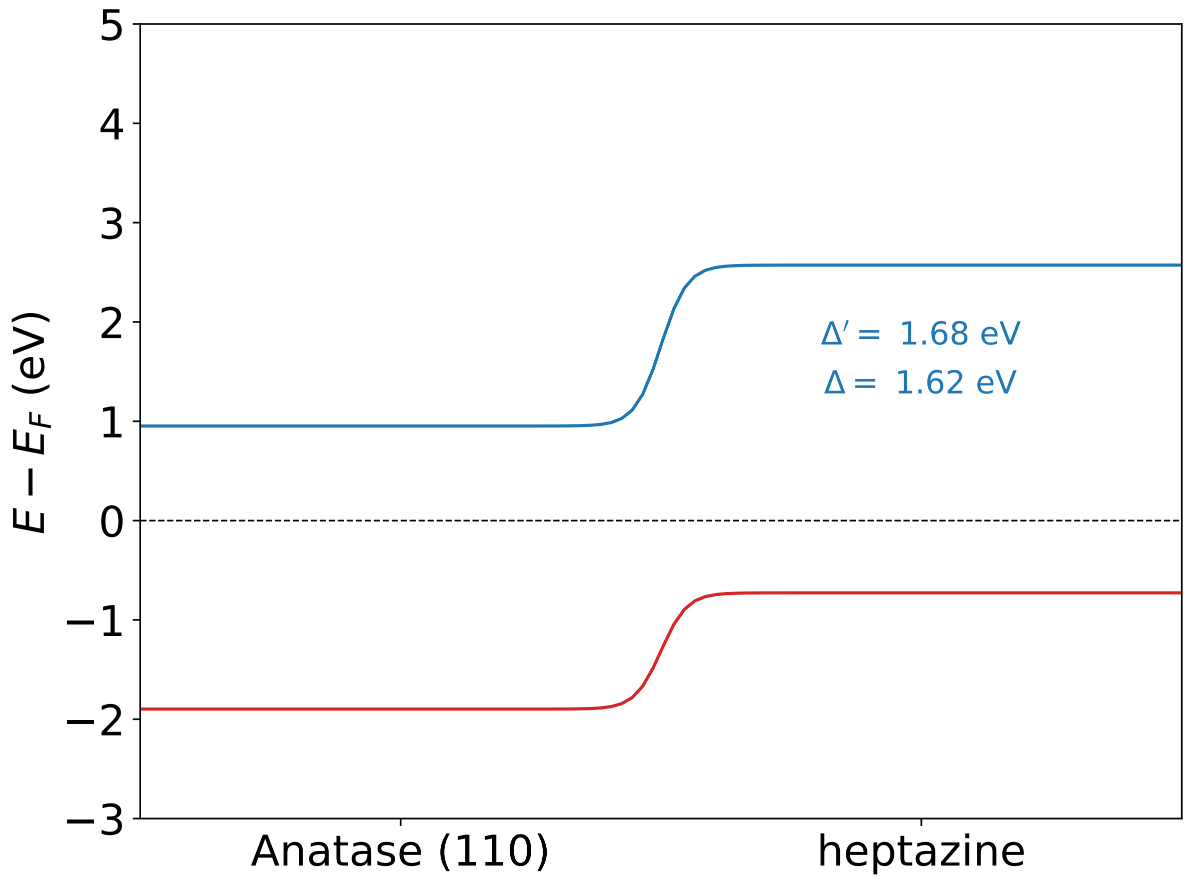
5
4
3
2
1
0
−1
−2
−3
$E - E_F$ (eV)
$\Delta' =$ 1.68 eV
$\Delta =$ 1.62 eV
Anatase (110)
heptazine

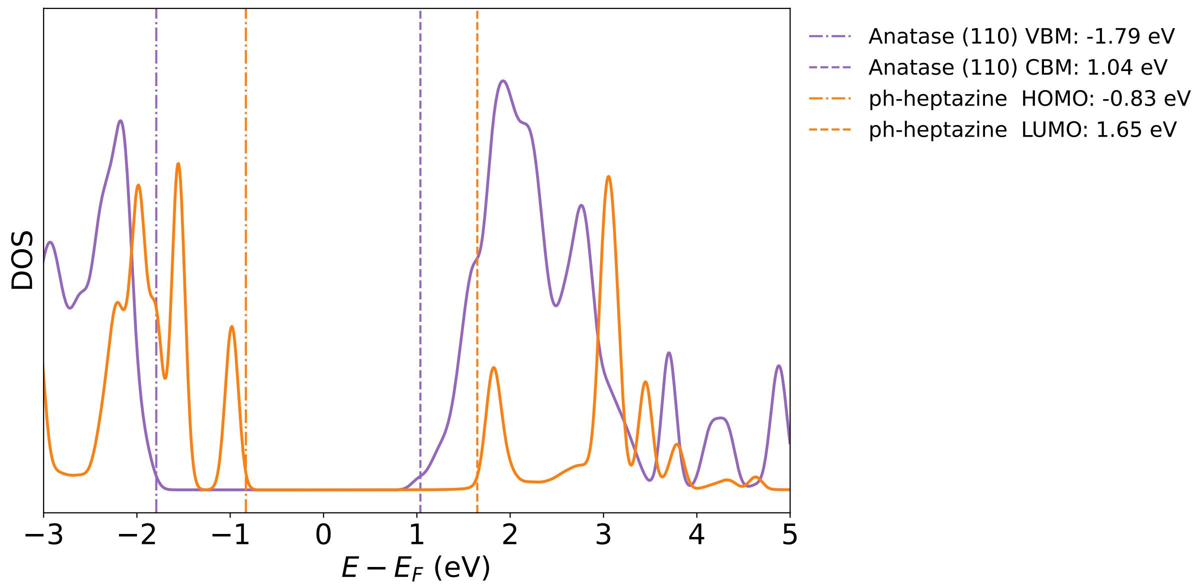
Anatase (110) VBM: -1.79 eV
Anatase (110) CBM: 1.04 eV
ph-heptazine HOMO: -0.83 eV
ph-heptazine LUMO: 1.65 eV
DOS
−3
−2
−1
0
1
2
3
4
5
$E - E_F$ (eV)

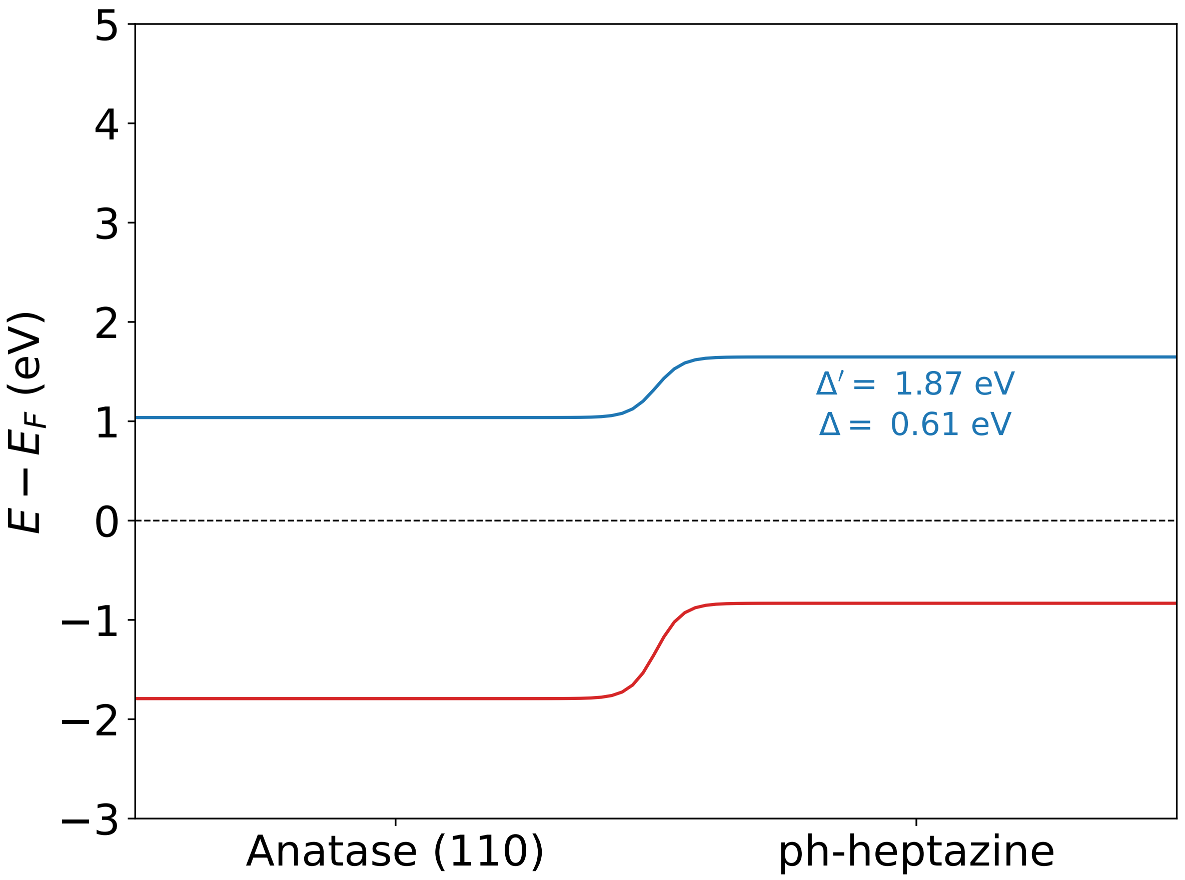
5
4
3
2
1
0
−1
−2
−3
$E - E_F$ (eV)
Δ′ = 1.87 eV
Δ = 0.61 eV
Anatase (110)
ph-heptazine

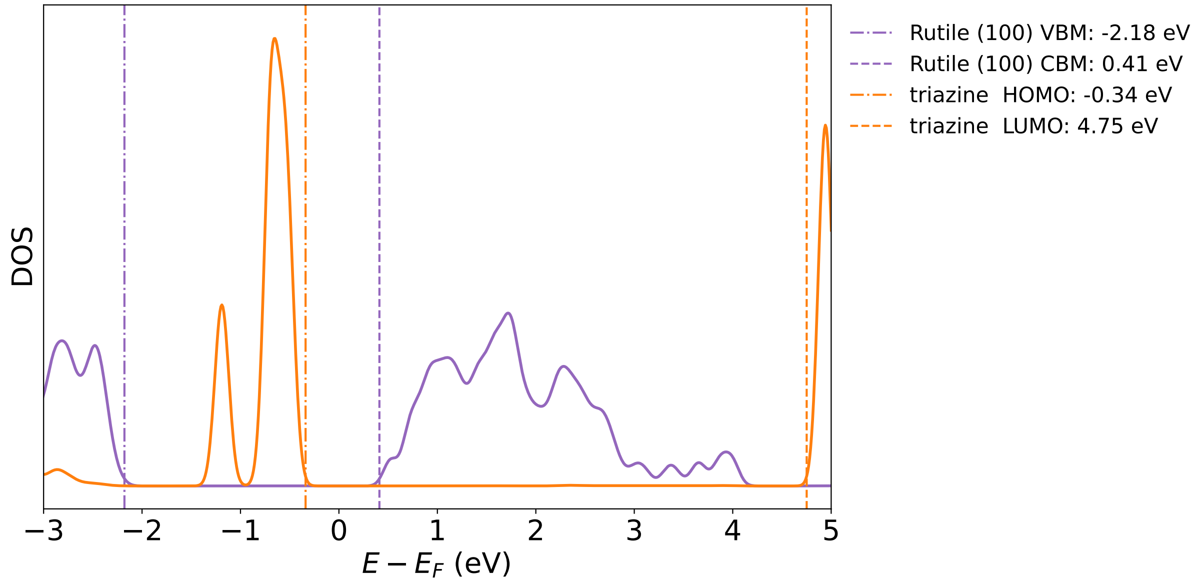
DOS
−3
−2
−1
0
1
2
3
4
5
$E - E_F$ (eV)
Rutile (100) VBM: -2.18 eV
Rutile (100) CBM: 0.41 eV
triazine HOMO: -0.34 eV
triazine LUMO: 4.75 eV

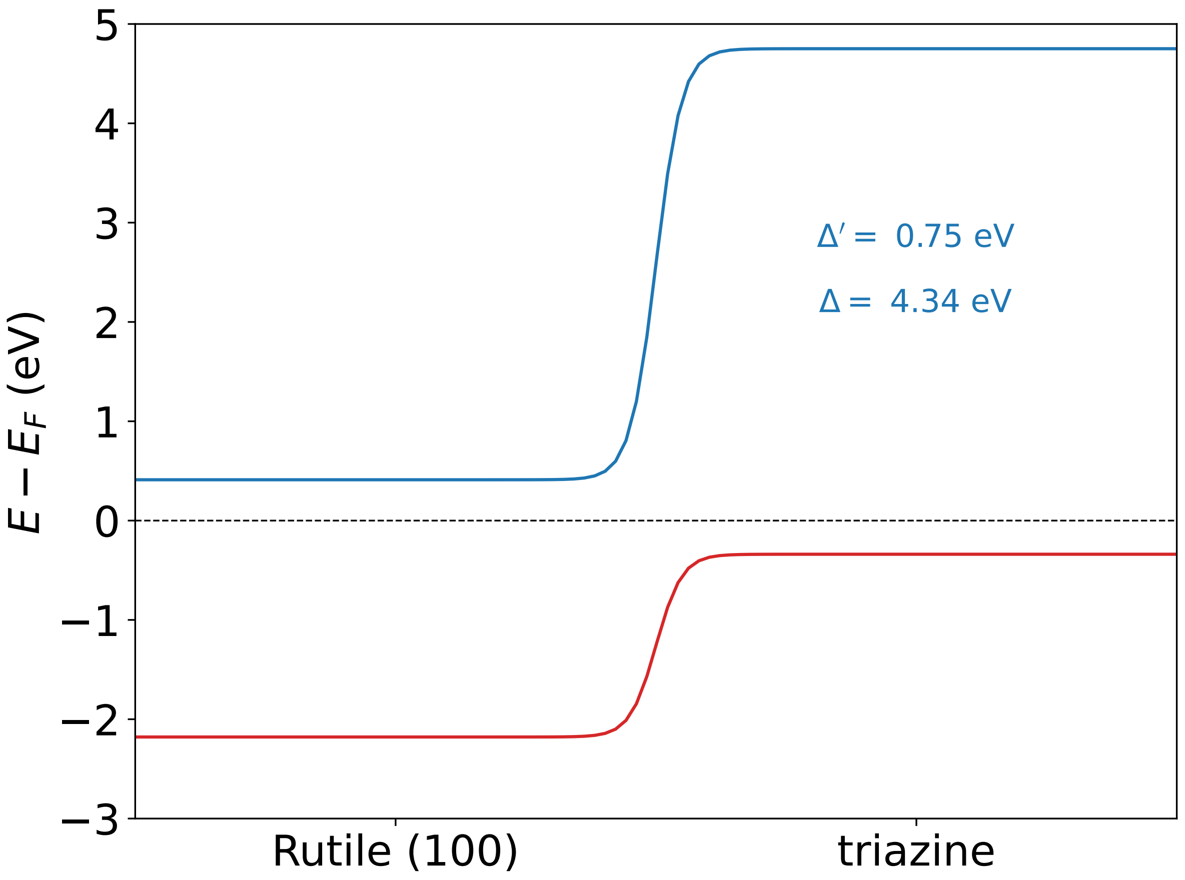

$E - E_F$ (eV)
$\Delta' =$ 0.75 eV
$\Delta =$ 4.34 eV
Rutile (100)
triazine

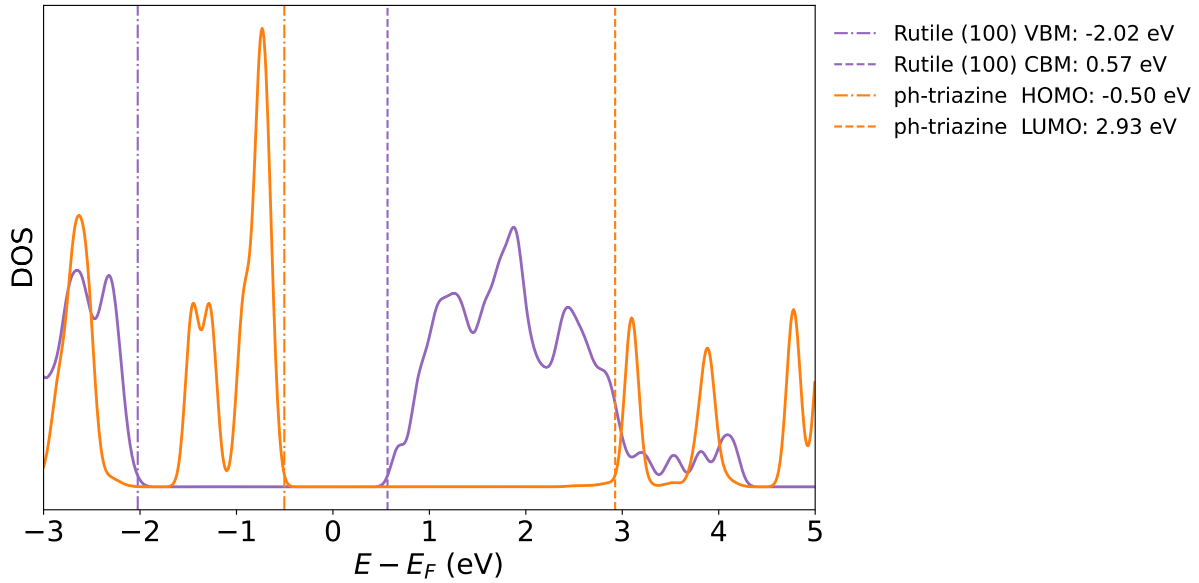

DOS
$E - E_F$ (eV)
Rutile (100) VBM: -2.02 eV
Rutile (100) CBM: 0.57 eV
ph-triazine HOMO: -0.50 eV
ph-triazine LUMO: 2.93 eV

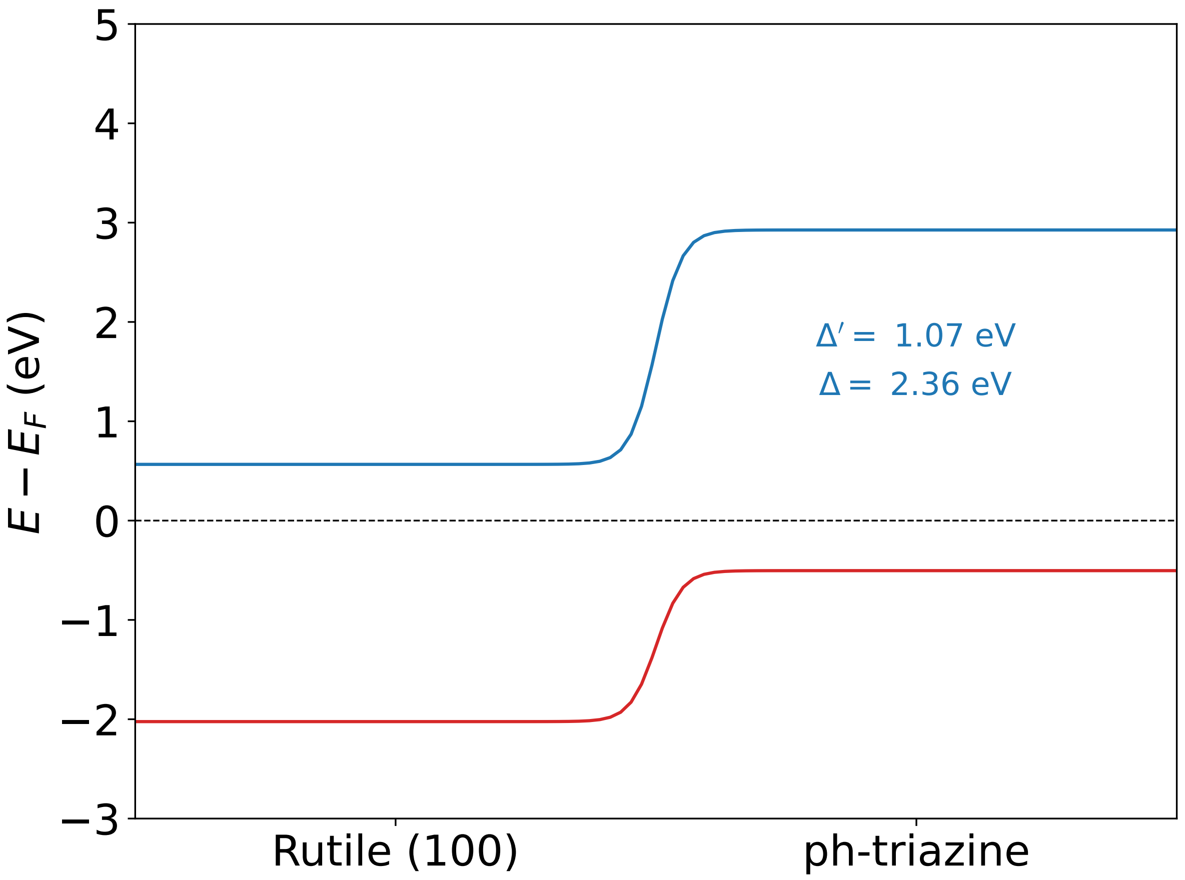
$E - E_F$ (eV)
Δ′ = 1.07 eV
Δ = 2.36 eV
Rutile (100)
ph-triazine

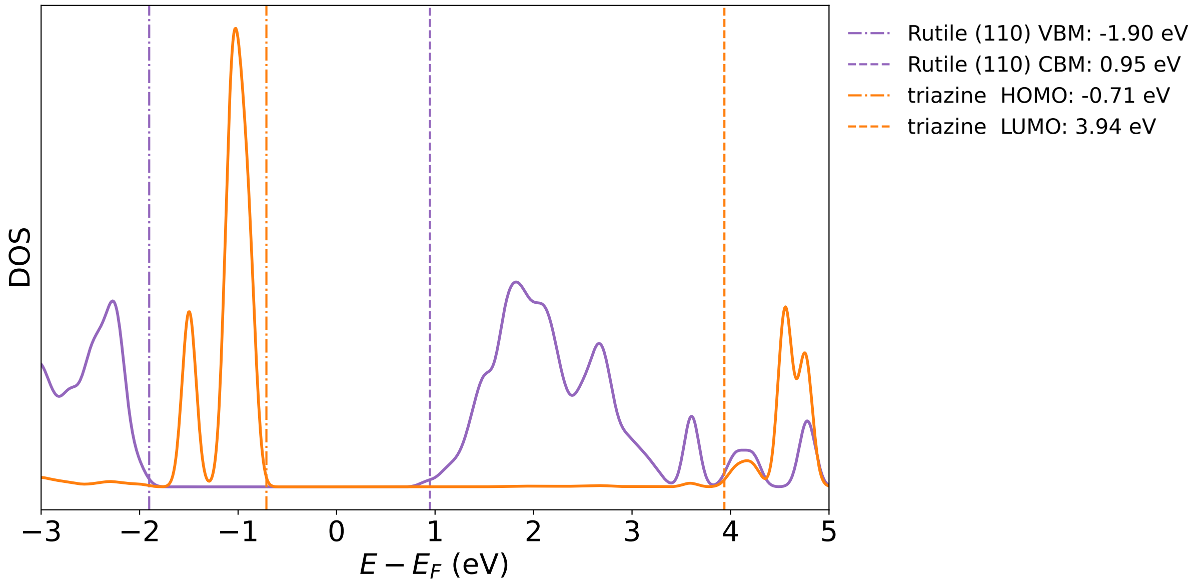
DOS
$E - E_F$ (eV)
Rutile (110) VBM: -1.90 eV
Rutile (110) CBM: 0.95 eV
triazine HOMO: -0.71 eV
triazine LUMO: 3.94 eV

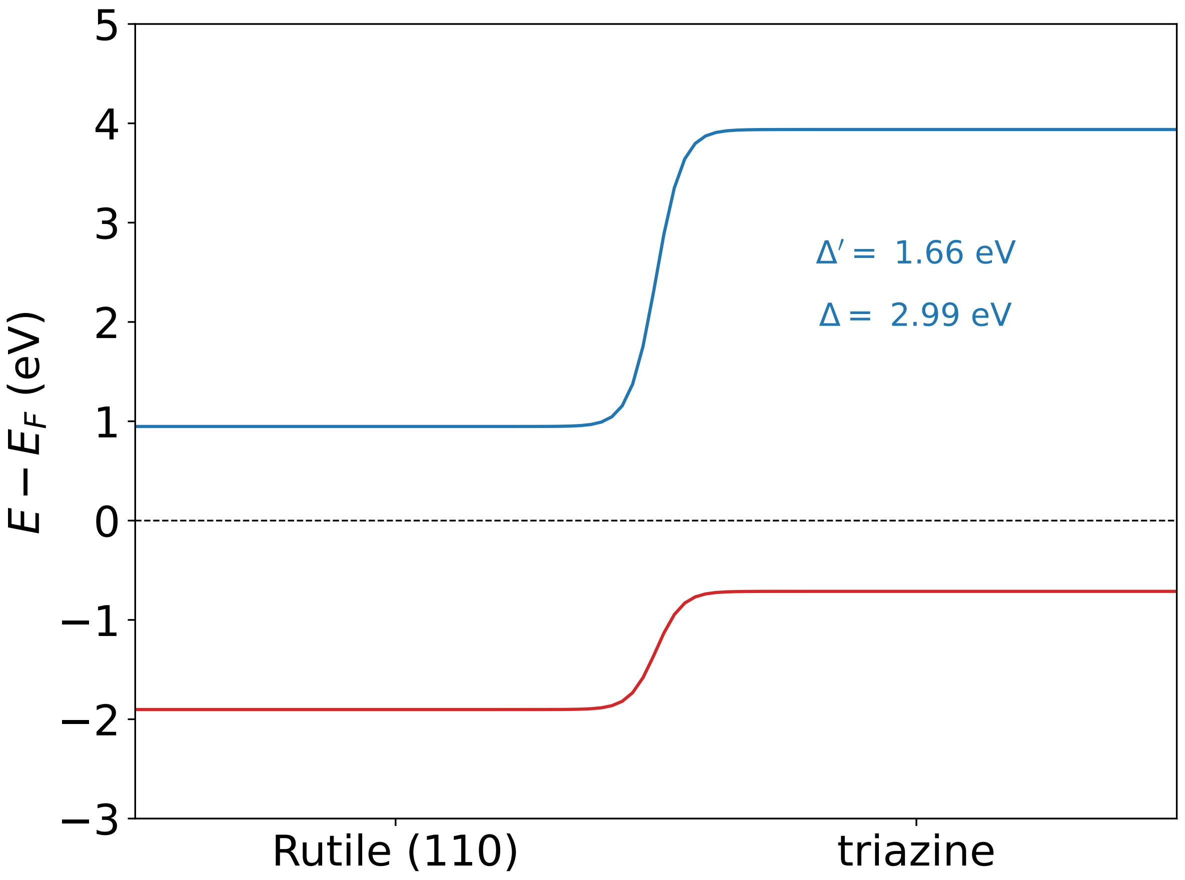
$\Delta' =$ 1.66 eV
$\Delta =$ 2.99 eV
$E - E_F$ (eV)
Rutile (110)
triazine

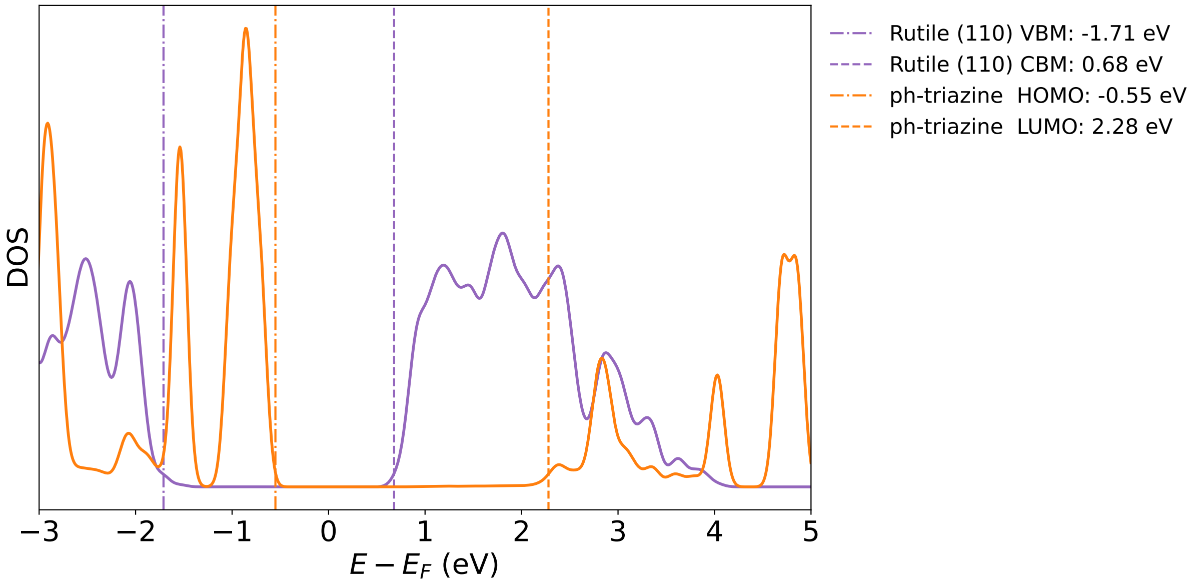
Rutile (110) VBM: -1.71 eV
Rutile (110) CBM: 0.68 eV
ph-triazine HOMO: -0.55 eV
ph-triazine LUMO: 2.28 eV
DOS
$E - E_F$ (eV)

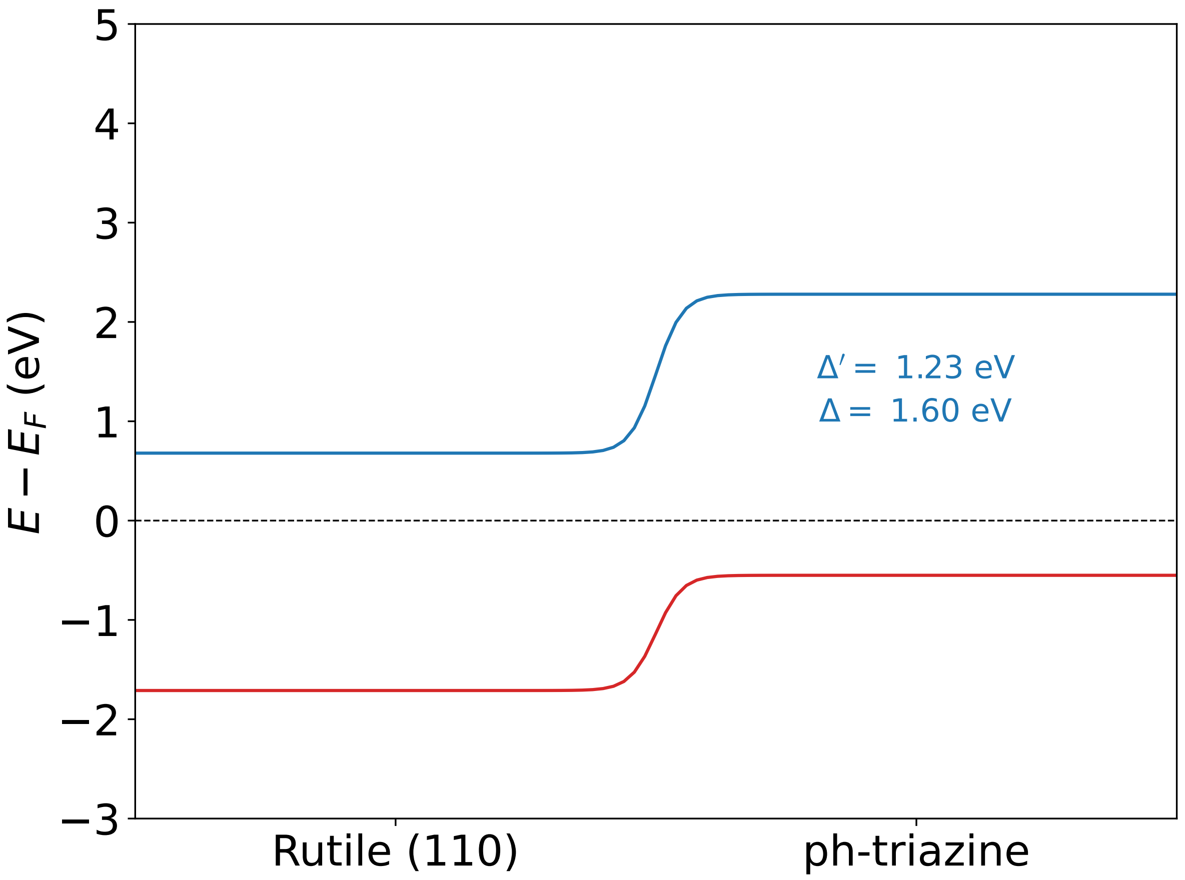

5
4
3
2
1
0
−1
−2
−3
$E - E_F$ (eV)
$\Delta' =$ 1.23 eV
$\Delta =$ 1.60 eV
Rutile (110)
ph-triazine

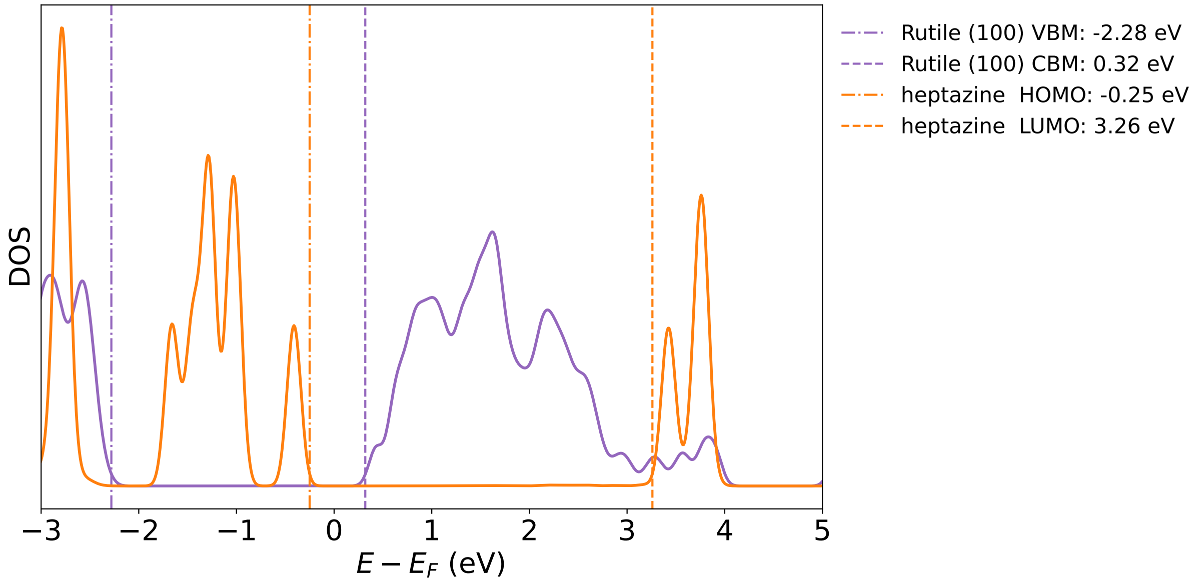

DOS
−3
−2
−1
0
1
2
3
4
5
$E - E_F$ (eV)
Rutile (100) VBM: -2.28 eV
Rutile (100) CBM: 0.32 eV
heptazine HOMO: -0.25 eV
heptazine LUMO: 3.26 eV

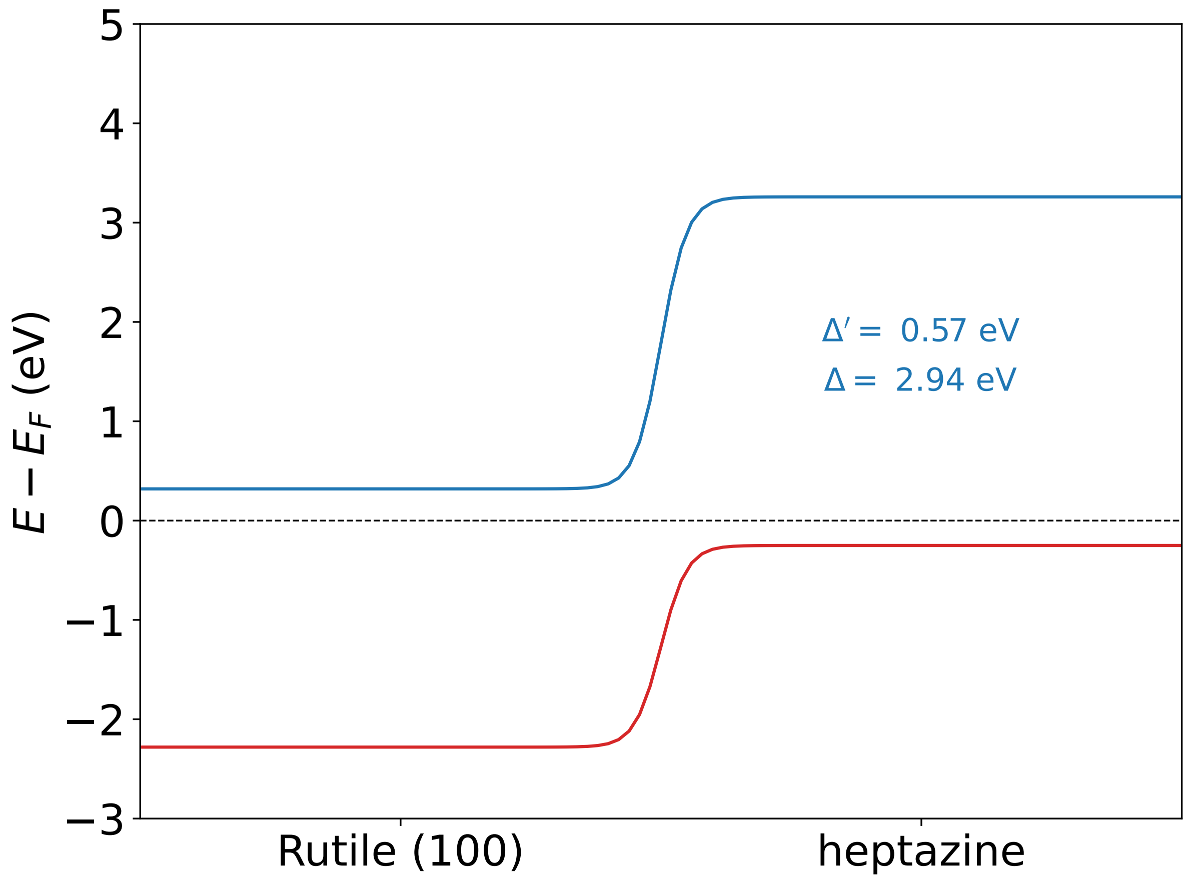
5
4
3
2
1
0
−1
−2
−3
$E - E_F$ (eV)
Δ′ = 0.57 eV
Δ = 2.94 eV
Rutile (100)
heptazine

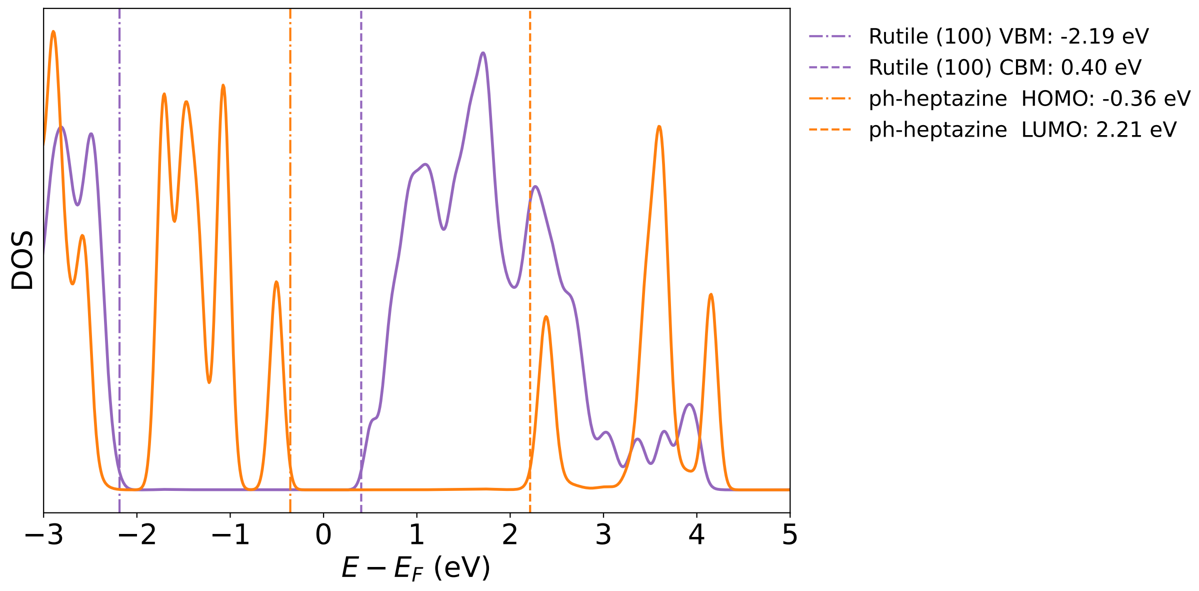
DOS
−3
−2
−1
0
1
2
3
4
5
$E - E_F$ (eV)
Rutile (100) VBM: -2.19 eV
Rutile (100) CBM: 0.40 eV
ph-heptazine HOMO: -0.36 eV
ph-heptazine LUMO: 2.21 eV

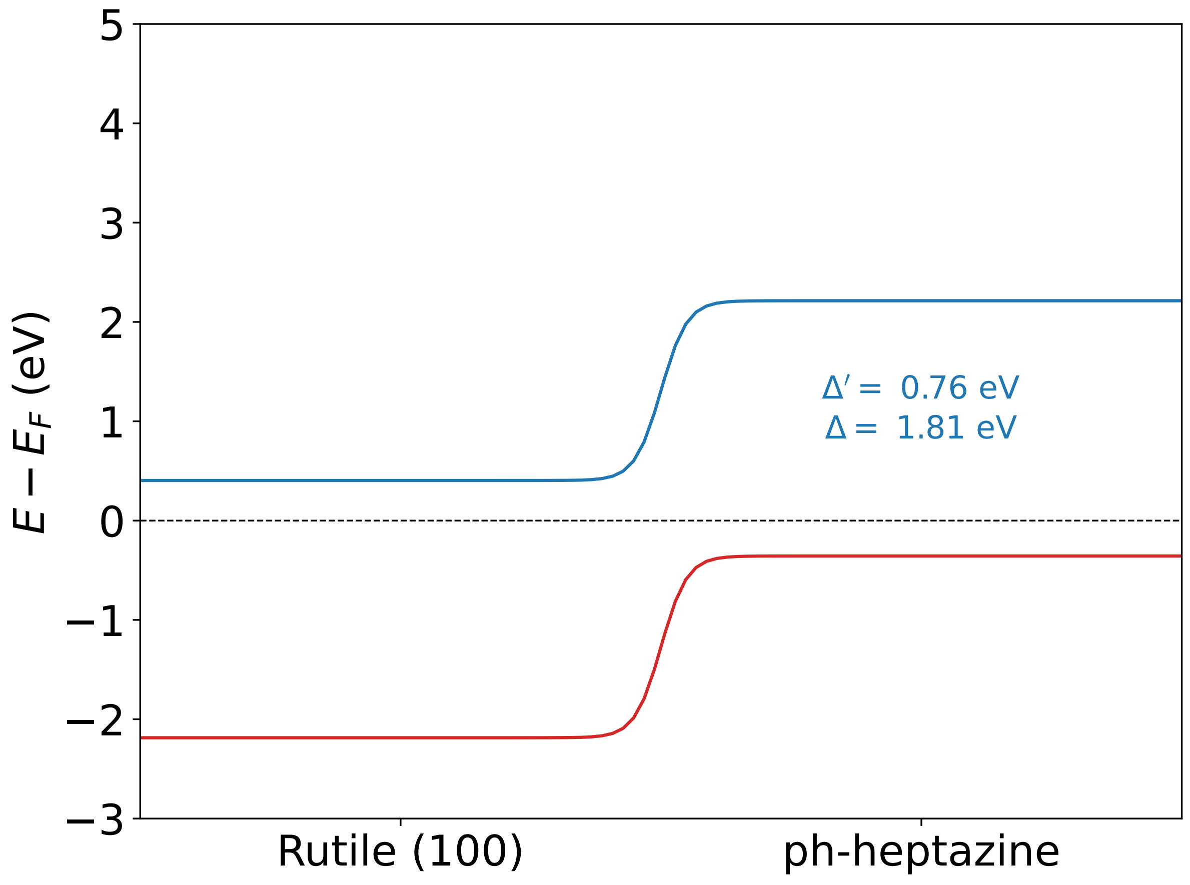

5
4
3
2
1
0
−1
−2
−3
E − E_F (eV)
Δ′ = 0.76 eV
Δ = 1.81 eV
Rutile (100)
ph-heptazine
